# Hepatic gene expression profiles during fed–fasted–refed state in mice

**DOI:** 10.3389/fgene.2023.1145769

**Published:** 2023-03-03

**Authors:** Nana Ji, Liping Xiang, Bing Zhou, Yan Lu, Min Zhang

**Affiliations:** ^1^ Department of Endocrinology and Metabolism, Qingpu Branch of Zhongshan Hospital affiliated to Fudan University, Shanghai, China; ^2^ Shanghai Key Laboratory of Diabetes Mellitus, Department of Endocrinology and Metabolism, Shanghai Diabetes Institute, Shanghai Clinical Centre for Diabetes, Shanghai Sixth People’s Hospital Affiliated to Shanghai Jiao Tong University School of Medicine, Shanghai, China; ^3^ Institute of Metabolism and Regenerative Medicine, Shanghai Sixth People’s Hospital Affiliated to Shanghai Jiao Tong University School of Medicine, Shanghai, China

**Keywords:** liver, fasting, refed, gene expression, microRNA, fed

## Abstract

**Background:** Regulation of nutrient status during fasting and refeeding plays an important role in maintaining metabolic homeostasis in the liver. Thus, we investigated the impact of the physiological Fed–Fast–Refed cycle on hepatic gene expression in nutrient-sensitive mice.

**Methods:** We performed transcriptomic analysis of liver samples in fed, fasted and refed groups of mice. Through mRNA-sequencing (RNA-Seq) and miRNA-Seq, we compared fasted and fed states (fasted *versus* fed cohort) as well as refed and fasted states (refed *versus* fasted cohort) to detect dynamic alterations of hepatic mRNA–miRNA expression during the fed–fasted–refed cycle.

**Results:** We found dozens of dysregulated mRNAs–miRNAs in the transition from fed to fasted and from fasted to refed states. Gene set enrichment analysis showed that gene expression of the two cohorts shared common pathways of regulation, especially for lipid and protein metabolism. We identified eight significant mRNA and three miRNA clusters that were up–downregulated or down–upregulated during the Fed–Fast–Refed cycle. A protein–protein interaction network of dysregulated mRNAs was constructed and clustered into 22 key modules. The regulation between miRNAs and target mRNAs was presented in a network. Up to 42 miRNA–mRNA-pathway pairs were identified to be involved in metabolism. In lipid metabolism, there were significant correlations between mmu-miR-296-5p and Cyp2u1 and between mmu-miR-novel-chr19_16777 and Acsl3.

**Conclusion:** Collectively, our data provide a valuable resource for the molecular characterization of the physiological Fed–Fast–Refed cycle in the liver.

## Introduction

Obesity has emerged as a global public health problem, and improvement of nutrient status and dietary interventions have been touted as potential remedies. To achieve resistance to environmental stresses and toxicity, fasting can bring cells and tissues into a protected state. It is reported that preoperative fasting can alleviate hepatic damage induced by ischemia/reperfusion injury (Amer et al., 2017). Nutrient status is regulated by highly variable molecular mechanisms and has an impact on metabolic homeostasis in the liver, particularly for glucose, lipid and energy metabolism (Koliaki and Roden, 2013; Jones, 2016). To gain a comprehensive understanding of molecular alterations in different nutrient statuses, we performed hepatic mRNA-sequencing (RNA-Seq) and miRNA-Seq during the physiological Fed–Fast–Refed cycle in mice.

Over the past decade, RNA-Seq has become an indispensable tool for transcriptome-wide analysis of differential gene expression and differential splicing of mRNAs, which interrogate global gene expression changes at the transcriptional level (Stark et al., 2019; Hong et al., 2020). miRNAs are a family of post-transcriptional gene repressors and are associated with the regulation of gene expression in metabolism (Lu and Rothenberg, 2018; Agbu and Carthew, 2021). To date, several microarray profiling studies have been performed to investigate the Fed–Fast–Refed cycle. However, most transcriptomic studies in the liver have focused on a single aspect of the cycle, such as transition from fed to fasted or from fasted to refed states (Chi et al., 2020; Hwangbo et al., 2020; Wahl and LaRocca, 2021), which might be inadequate. Our study investigated the Fed–Fast–Refed cycle comprehensively and combined RNA-Seq with miRNA-Seq analysis.

We performed a systematic evaluation of hepatic genome-wide mRNA and miRNA expression through RNA-Seq and miRNA-Seq in mice in fed, fasted and refed states. We compared mRNA–miRNA expression during the transition from fed to fasted and from fasted to refed states. We analyzed alterations in mRNAs–miRNAs and related pathways in fasted *versus* fed and refed *versus* fasted cohorts. We detected significant mRNA and miRNA clusters that were upregulated and subsequently downregulated (up–down) or downregulated and subsequently upregulated (down–up) during the Fed–Fast–Refed cycle. A regulatory network including protein–protein interaction (PPI), miRNA–mRNA and miRNA–mRNA pathways was established for further analysis in the Fed–Fast–Refed cycle. We aimed to provide novel insights into the molecular characteristics of the physiological impact of the Fed–Fast–Refed cycle in the liver.

## Materials and methods

### Animal experiments

The animal procedures were approved by the Animal Experiment Ethics Committees of Shanghai Jiao Tong University School of Medicine. Wild-type male C57BL/6J mice aged 8 weeks were purchased from Shanghai Laboratory Animal Company (SLAC, Shanghai, China). All mice were housed at 21°C ± 1°C with a humidity of 55% ± 10% and 12-h light/12-h dark cycle in a specific-pathogen-free facility. After 2 weeks of acclimatization, mice were divided into three groups. The fasted group was fed a regular diet with subsequent fasting for 24 h. The refed group underwent 24 h fasting and was refed a fixed-calorie meal for 2 h. Mice were anesthetized with sodium pentobarbital (Nembutal, 80 mg/kg, i.p.) and killed during the fasted and refed states. Liver tissues were harvested and snap-frozen in liquid nitrogen for further analysis.

### RNA-Seq and data processing

Total RNA was extracted using TRIzol reagent (Invitrogen, Carlsbad, CA, United States). A total of 3 µg RNA per sample was used as input material for the RNA sample preparations. Sequencing libraries were generated using the NEBNext^®^ Ultra™ RNA Library Prep Kit for Illumina^®^ (NEB, United States), and index codes were added to attribute sequences to each sample. Library quality was assessed by Agilent Bioanalyzer 2100 system. The sequencing libraries were sequenced on an Illumina Hiseq2500/X platform. For mRNA sequencing, cuffdiff software (part of cufflinks) was used to obtain FPKM as the expression profiles of mRNA and differentially expressed mRNAs were calculated based on log(FPKM+1) with *p* < 0.05 and |Fold Change|≥2 used as the cutoff values. For miRNA sequencing, limma R package (http://bioconductor.org/packages/release/bioc/html/limma.html) (Ritchie et al., 2015) was used to obtain scaled raw counts, and differentially expressed miRNAs were identified with *p* < 0.05 and |Fold Change|≥2 used as the cutoff values. Pearson coefficient *r* ^2^ values were calculated based on FPKM values and raw counts in RNA-Seq and miRNA-Seq, respectively. Principal component analysis (PCA) was performed by R ggfortify package (http://bioconductor.org/packages/release/bioc/html/ggfortify.html). Heatmaps were plotted by applying R pheatmap package (http://bioconductor.org/packages/release/bioc/html/pheatmap.html).

### Functional enrichment analysis

Gene Ontology (GO) annotation (Ashburner et al., 2000) includes three categories: biological process, cellular compartment and molecular function. Biological process of GO and Kyoto Encyclopedia of Genes and Genomes (KEGG) pathway enrichment analysis (Kanehisa and Goto, 2000) was performed by DAVID (Sherman et al., 2022) online tool (https://david.ncifcrf.gov/tools.jsp) with dysregulated mRNAs. Gene set enrichment analysis (GSEA) (Subramanian et al., 2005) was performed based on the log2(Fold Change) of mRNA by R clusterProfiler package (Yu et al., 2012) (http://bioconductor.org/packages/release/bioc/html/clusterProfiler.html). *p* < 0.05 and gene number in one term ≥2 was identified as significant enrichment.

### Expression trend analysis

We obtained the union set of dysregulated mRNAs–miRNAs in the fasted *versus* fed and refed *versus* fasted cohorts, which were considered as mRNAs–miRNAs pairs in the Fed–Fast–Refed cycle. Trend cluster analysis was performed with these mRNAs–miRNAs to explore expression trends in the Fed–Fast–Refed cycle based on the R Mfuzz package (Kumar and Futschik, 2007) (http://bioconductor.org/packages/release/bioc/html/Mfuzz.html). Membership ≥0.3 was used as the cutoff value. For each cluster, large membership values indicated that the genes were in accordance with the expression trend cluster. Next, clusters with similar expression trends were merged. We focused on trend clusters that were upregulated and subsequently downregulated (up–down) and downregulated and subsequently upregulated (down–up) in the Fed–Fast–Refed cycle.

### PPI network analysis

Based on the STRING (http://www.string-db.org/) dataset (von Mering et al., 2003), we predicted proteins encoded by dysregulated genes up–downregulated or down–upregulated in the Fed–Fast–Refed cycle and created a PPI network, which was visualized with cytoscape (http://chianti.ucsd.edu/cytoscape-3.4.0//) (Nangraj et al., 2020). The PPI score was set as 0.7, which was considered as high confidence. CytoNCA (http://apps.cytoscape.org/apps/cytonca) was applied to detect hub proteins through ranking Degree Centrality. MCODE (http://apps.cytoscape.org/apps/mcode) (Bader and Hogue, 2003) was applied to calculate key modules in the PPI network (Degree Cutoff = 2, Node Score Cutoff = 0.2, K-core = 2 and Max.Depth = 100). KEGG pathway enrichment analysis of key modules was performed with R clusterProfiler package (http://bioconductor.org/packages/release/bioc/html/clusterProfiler.html) (Kanehisa and Goto, 2000). *p*.adjust<0.05 was identified as enrichment significant.

### miRNA–mRNA regulation network analysis

miRanda (http://www.mircorna.org/) was applied to predict potential target mRNAs of miRNA. A score ≥140 and energy ≤−20 were set as cutoff values. We focused on dysregulated mRNAs–miRNAs in the Fed–Fast–Refed cycle and obtained miRNA–mRNA pairs through prediction by miRanda. miRNA–mRNA pairs were selected to construct an miRNA–mRNA regulatory network.

### miRNA–mRNA-pathway regulation analysis

A Sankey diagram was established between miRNA–mRNA pairs and mRNA-pathway pairs involved in metabolism. Significant correlation with *p* < 0.05 between miRNA and mRNA expression was presented with scatter plots.

## Results

### RNA-Seq and miRNA-Seq data validation

**FIGURE 1:**
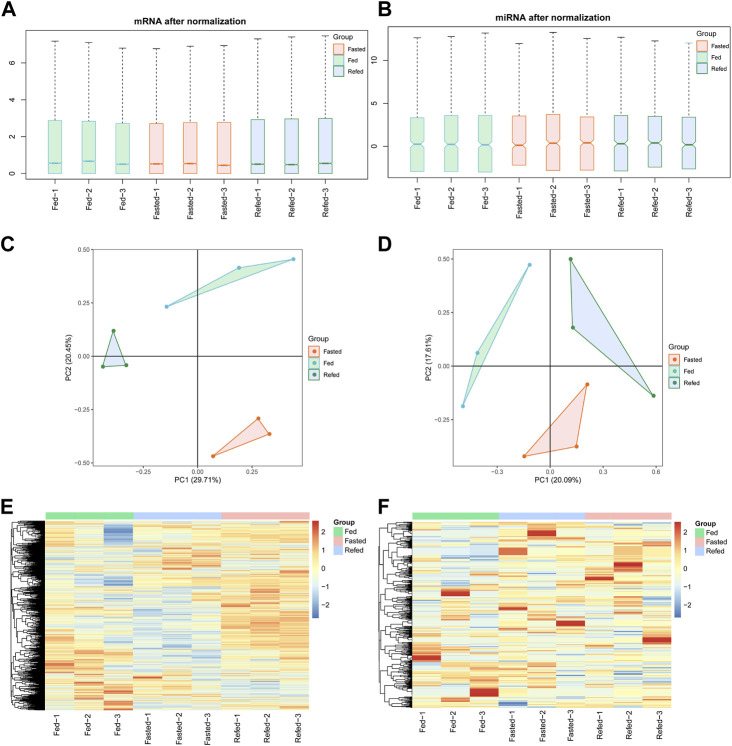
The reproductivity and reliability of mRNA-Seq and miRNA-Seq datasets. **(A,B)** Boxplot of mRNA **(A)** and miRNA **(B)** expression after normalization in fed, fasted and refed groups. **(C,D)** PCA analysis showing normalized expression of mRNA **(C)** and miRNA **(D)** in fed, fasted and refed groups after batch-effect correction. **(E,F)** Heatmap showing mRNA € and miRNA **(F)** expression patterns between fed, fasted and refed groups after batch-effect correction.

The mouse model for Fed–Fast–Refed cycle was constructed. As expected, blood glucose levels were reduced in the fasted state and increased in the refed state (Supplementary Figure S1A). Besides, expression levels of gluconeogenic (PEPCK and G6Pase) and lipogenic genes (SREBP-1c, Fasn, Scd1, and Acc1) confirmed that gluconeogenesis was induced by fasting and lipogenesis was increased by refeeding, respectively (Supplementary Figures S1B, S1C). Then, mouse hepatic genome-wide mRNA and miRNA expression was profiled using RNA-Sequencing and miRNA-Sequencing, respectively. Details of the study groups are listed in Table 1. The fed, fast and refed groups had three replicates each. mRNA–miRNA expression density plots in the fed, fasted and refed groups are presented in Supplementary Figures S2A, S2B. We demonstrated the reproducibility and reliability of mRNA–miRNA expression profiles. Correlation analysis showed that mRNA–miRNA expression reads were correlated well between different samples (Supplementary Figures S2C, S2D). Pearson correlation *r* ^2^ values between all samples in the three groups are shown in Supplementary Figures S2C, S2D. Box plots based on normalized mRNA–miRNA expression reads after batch-effect correction by interquartile range are shown in Figures 1A, B. PCA of mRNA–miRNA expression profiles showed that samples within each group were close, while samples between different groups were separated (Figures 1C, D). Hierarchical clustering analysis showed that samples in each group clustered together (Figures 1E, F). These data demonstrated that all of the RNA-Seq and miRNA-Seq results were reproducible and reliable for downstream analysis.

**TABLE 1:** Groups of mice used in this study.

Group	Strain	Treatment	Number of samples (N)	Profile
1	C57BL/6J	Fed	3	mRNA-Seq/miRNA-Seq
2	C57BL/6J	Fasted	3	mRNA-Seq/miRNA-Seq
3	C57BL/6J	Refed 2 h	3	mRNA-Seq/miRNA-Seq

### Differential expression analysis of RNA-Seq and miRNA-Seq

**FIGURE 2:**
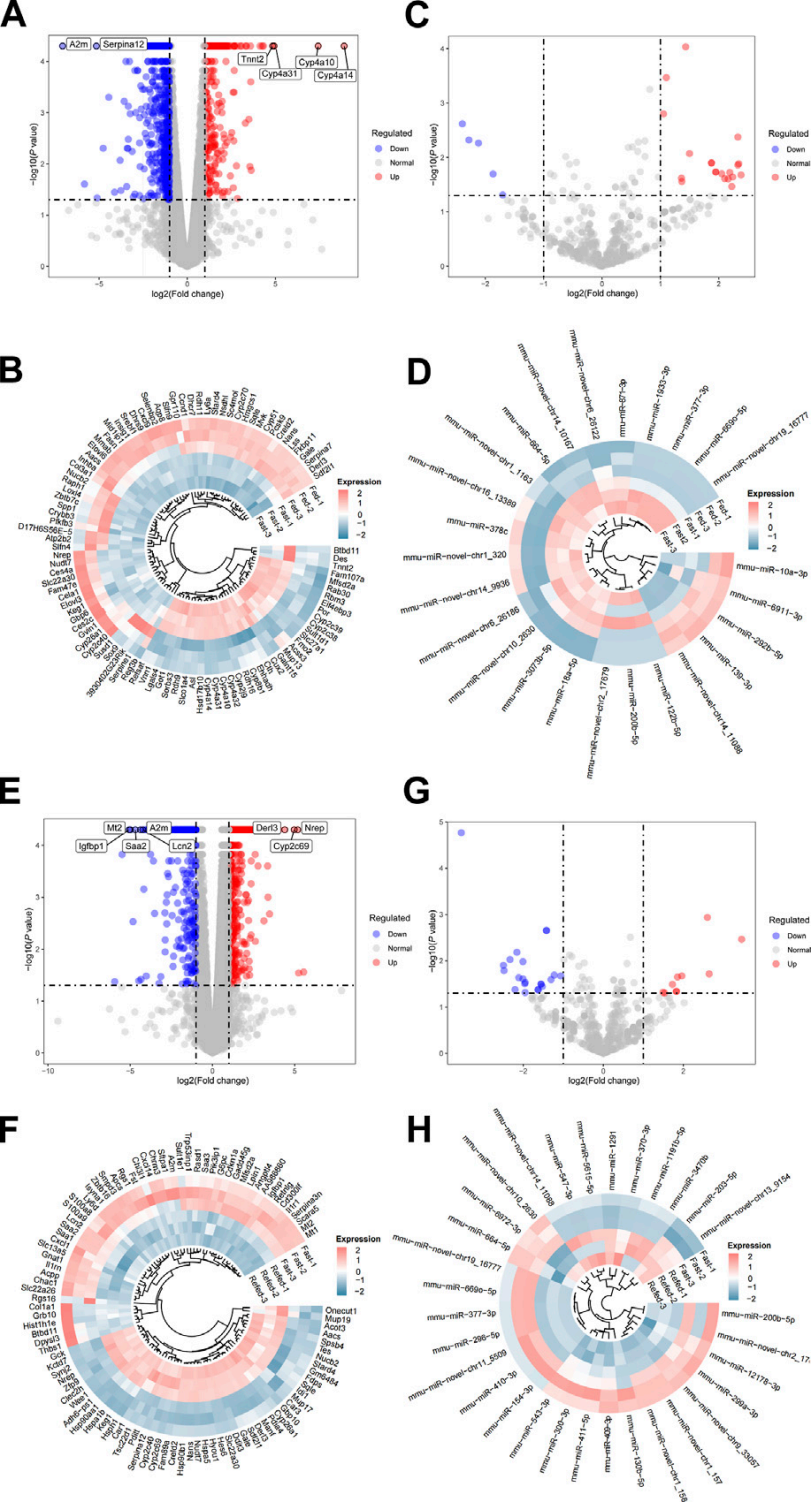
Differential expression analysis of RNA-Seq and miRNA-Seq datasets. **(A,B)** Hepatic DEGs during the transition from fed to fasted in mice. **(A)** Volcano plot of DEGs in the livers of mice in fasted compared with fed state. The top DEGs are labeled as indicated. **(B)** Heatmap showing expression patterns between top 100 DEGs in the liver of mice in fasted compared with fed state after batch-effect correction. **(C,D)** Hepatic dysregulated miRNAs during the transition from fed to fasted in mice. **(C)** Volcano plot of dysregulated miRNAs in the liver of mice in fasted compared with fed state. **(D)** Heatmap showing expression patterns between dysregulated miRNAs in the liver of mice in fasted compared with fed state after batch-effect correction. **(E,F)** Hepatic DEGs during the transition from fasted to refed state. **(E)** Volcano plot of DEGs in the liver of mice in refed compared with fasted state. **(F)** Heatmap showing expression patterns between top 100 DEGs in the liver of mice in refed compared with fasted state after batch-effect correction. **(G,H)** Hepatic dysregulated miRNAs during the transition from fasted to refed state. **(G)** Volcano plot of dysregulated miRNAs in the liver of mice in refed compared with fasted state. **(H)** Heatmap showing expression patterns between dysregulated miRNAs in the liver of mice in refed compared with fasted state after batch-effect correction.

To detect dynamic alterations of hepatic genome-wide mRNA and miRNA expression during the Fed–Fast–Refed cycle, comparisons were made between fasted and fed states (fasted *versus* fed cohort) as well as refed and fasted state (refed *versus* fasted cohort) using RNA-Seq and miRNA-Seq. *p* < 0.05 and fold change>2 were set as the threshold for identifying dysregulated mRNA and miRNAs. Initially, we investigated the fasted *versus* fed cohort. For RNA-Seq, the liver underwent dramatic changes in gene expression during transition from fed to fasted state, with a total of 874 differentially expressed genes (DEGs); of which, 291 were upregulated and 583 were downregulated (Figure 2A). The most significantly upregulated genes included those encoding Cyp4a14, Cyp4a10, Cyp4a31, and Tnnt2, while the most significantly downregulated genes included those encoding A2m and Serpina12 (Figure 2A). The top 100 significantly dysregulated genes in the fasted *versus* fed cohort are presented in the circle heatmap plot (Figure 2B). In addition, we undertook intersection analysis of our RNA-Seq analysis with a public dataset (GSE107787), in which mice were fasted 20 h. As a result, a total of 298 DEGs were found between both screening, of which 129 were upregulated and 169 were downregulated (Supplementary Document S1). For miRNA-Seq, we identified 25 dysregulated miRNAs in the liver of mice in the fasted state compared with fed state; of which, 20 were upregulated and five were downregulated (Figure 2C). These differentially expressed miRNAs are shown in the circle heatmap plot (Figure 2D).

In the refed *versus* fasted cohort, for mRNA-Seq, 1,048 genes were differentially expressed in the refed state compared with fasted state; among which, 698 were upregulated and 350 were downregulated (Figure 2E). The most significantly upregulated genes included those encoding Nrep, Cyp2c69, and Derl3, while the most significantly downregulated genes included those encoding Mt2, Igfbp1, Saa2, Lcn2, and A2m (Figure 2E). The top 100 significantly dysregulated genes in the refed *versus* fasted cohort are presented in the circle heatmap plot (Figure 2F). We also undertook intersection analysis of our analysis with a public dataset (GSE137385), in which mice were refed 3 h with low-fat diet after fasting. As a result, a total of 157 DEGs were found between both screening, of which 87 were upregulated and 70 were downregulated (Supplementary Document S2). These comparisons would inform on which gene expression sets change robustly enough across platforms and somewhat differing experimental conditions. For miRNA-Seq, we identified 32 differentially expressed miRNAs in the liver of mice in the refed state compared with fasted state; of which, 10 were upregulated and 22 were downregulated (Figure 2G). These differentially expressed miRNAs are shown in the circle heatmap plot (Figure 2H). Numbers of differentially expressed mRNAs and miRNAs detected are summarized in Table 2.

**TABLE 2:** Numbers of differentially expressed genes and miRNAs detected.

		mRNA	miRNA
Fasted *versus* fed	Up	291	20
	Down	583	5
	Total	874	25
Refed *versus* fasted	Up	698	10
	Down	350	22
	Total	1,048	32

### KEGG signaling enrichment analysis of mRNA expression profile based on GSEA

**FIGURE 3:**
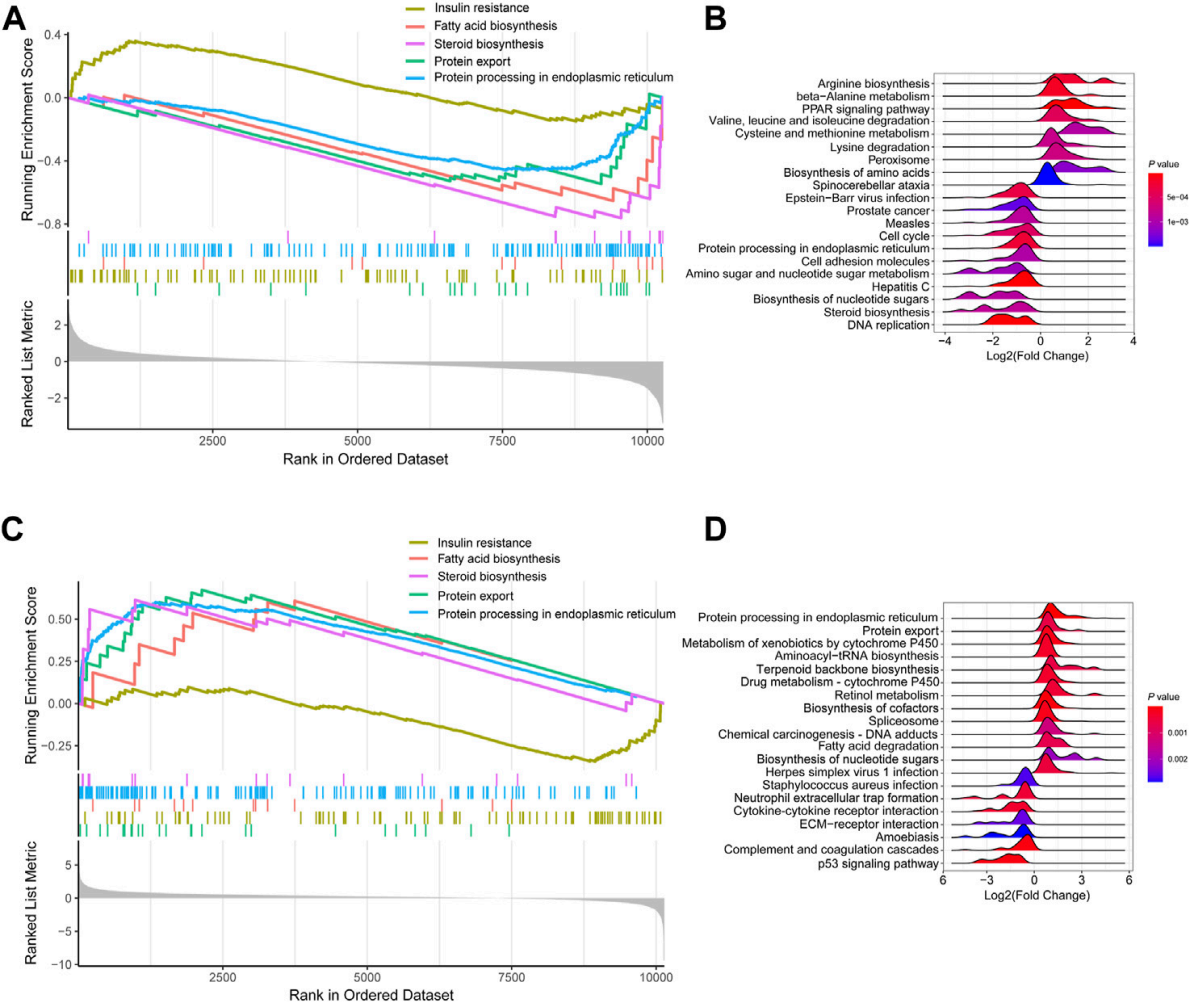
KEGG signaling enrichment analysis of mRNA expression profile based on GSEA. **(A,B)** KEGG signaling enrichment analysis of mRNA expression profile in fasted *versus* fed cohort. **(A)** Selected GSEA results of mRNA expression profile in fasted *versus* fed cohort. Running enrichment score and ranked list are presented. **(B)** Ridge plot listed the top 20 pathways in fasted *versus* fed cohort. **(C,D)** KEGG signaling enrichment analysis of mRNA expression profile in refed *versus* fasted cohort. **(C)** Selected GSEA results of mRNA expression profile in refed *versus* fasted cohort. Running enrichment score and ranked list are presented. **(D)** Ridge plot listed the top 20 pathways in refed *versus* fasted cohort.

KEGG signaling enrichment analysis based on GSEA was performed with log2(Fold Change) of all mRNAs in the fasted *versus* fed and refed *versus* fasted cohorts. In the fasted *versus* fed cohort, GSEA demonstrated that mRNAs were mainly mapped to 66 KEGG pathways; of which, 29 showed a trend to be upregulated, while 37 showed a trend to be downregulated (Supplementary Tables S1, S2). In particular, pathways such as insulin resistance showed a trend to be upregulated (Figure 3A), and pathways including fatty acid biosynthesis, steroid biosynthesis, protein export and protein processing in the endoplasmic reticulum (ER) showed a trend to be downregulated (Figure 3A). The top 20 pathways according to *p*-value were listed in the ridge plot, which showed the distribution of log2(Fold Change) of genes enriched in each pathway (Figure 3B). The top 20 pathways included nine pathways in which enriched genes were mainly upregulated and 11 in which enriched genes were mainly downregulated (Figure 3B). In the refed *versus* fasted cohort, GSEA demonstrated that mRNAs were mainly mapped to 70 KEGG pathways, of which, 33 showed a trend to be upregulated, and 37 showed a trend to be downregulated (Supplementary Tables S3, S4). Pathways including fatty acid biosynthesis, steroid biosynthesis, protein export and protein processing in the ER showed a trend to be upregulated (Figure 3C), and insulin resistance showed a trend to be downregulated (Figure 3C). The pathway results in the refed *versus* fasted cohort were in contrast with those in the fasted *versus* fed cohort. The top 20 pathways according to *p*-value are listed in the ridge plot (Figure 3D). The top 20 pathways included 13 in which enriched genes were mainly upregulated and seven in which enriched genes were mainly downregulated (Figure 3D).

### Expression trend analysis

**FIGURE 4:**
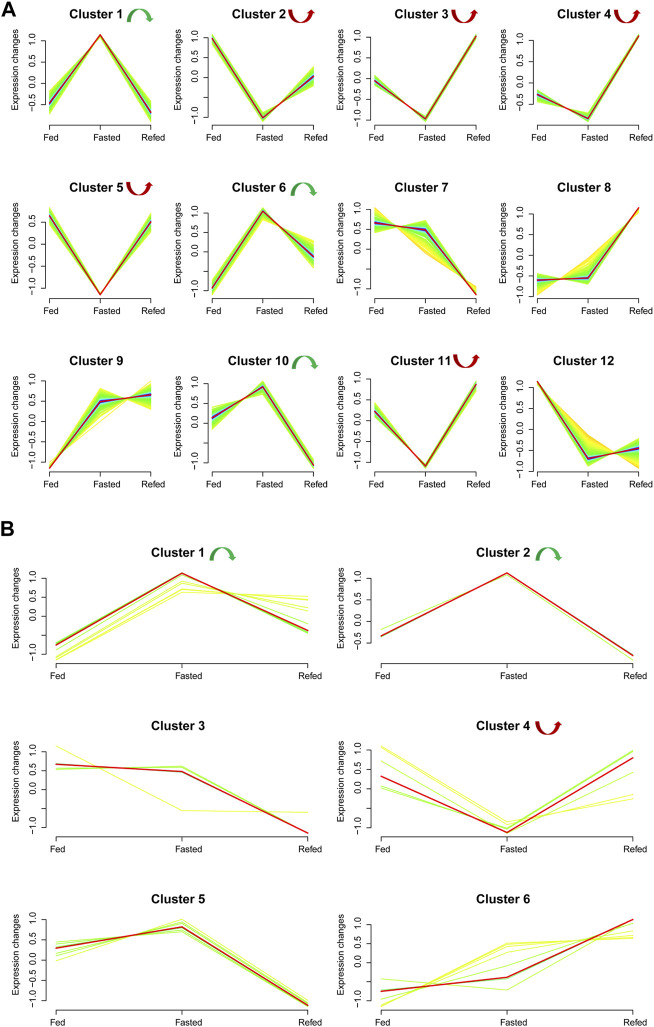
Expression trend analysis. **(A)** The results of expression trend analysis based on the union set of dysregulated mRNAs in fasted *versus* fed and refed *versus* fasted cohorts. Twelve mRNA clusters were detected. With a member.ship ≥0.3 used as the cutoff value, we identified eight significant mRNA clusters in the Fed–Fast–Refed cycle; three were upregulated and subsequently downregulated (1, 6 and 10) and five were downregulated and subsequently upregulated (2–5 and 11). **(B)** Expression trend analysis based on the union set of dysregulated miRNAs in fasted *versus* fed and refed *versus* fasted cohorts. Six miRNA clusters were detected. With a member.ship ≥0.3 used as the cutoff value, we identified three significant miRNA clusters in the Fed–Fast–Refed cycle; two were upregulated and subsequently downregulated (1 and 2) and one was downregulated and subsequently upregulated (4).

We analyzed the union set of dysregulated mRNAs/miRNAs in the fasted *versus* fed and refed *versus* fasted cohorts, resulting in 1,579 mRNAs and 48 miRNAs (Supplementary Documents S3, S4). Based on these dysregulated mRNAs–miRNAs in the union set, trend cluster analysis was performed using R Mfuzz package to detect mRNAs–miRNAs expression trends in the Fed–Fast–Refed cycle, and 12 mRNA clusters were obtained (Figure 4A). Details are summarized in Supplementary Document S3. Member.ship≥0.3 was used as the cutoff value. In the Fed–Fast–Refed cycle, we identified eight significant mRNA clusters including three (1, 6 and 10; 170 mRNAs) that were upregulated with subsequent downregulation, and five significant mRNA clusters (2–5 and 11; 474 mRNAs) that were downregulated with subsequent upregulation. Six miRNA clusters were obtained (Figure 4B). Details are summarized in Supplementary Document S4. In the Fed–Fast–Refed cycle, we identified three significant miRNA clusters including two (1 and 2; 11 miRNAs) that were upregulated with subsequent downregulation, and cluster 4 that was downregulated and subsequently upregulated.

### Functional enrichment of dysregulated mRNAs in significant mRNA clusters

**FIGURE 5:**
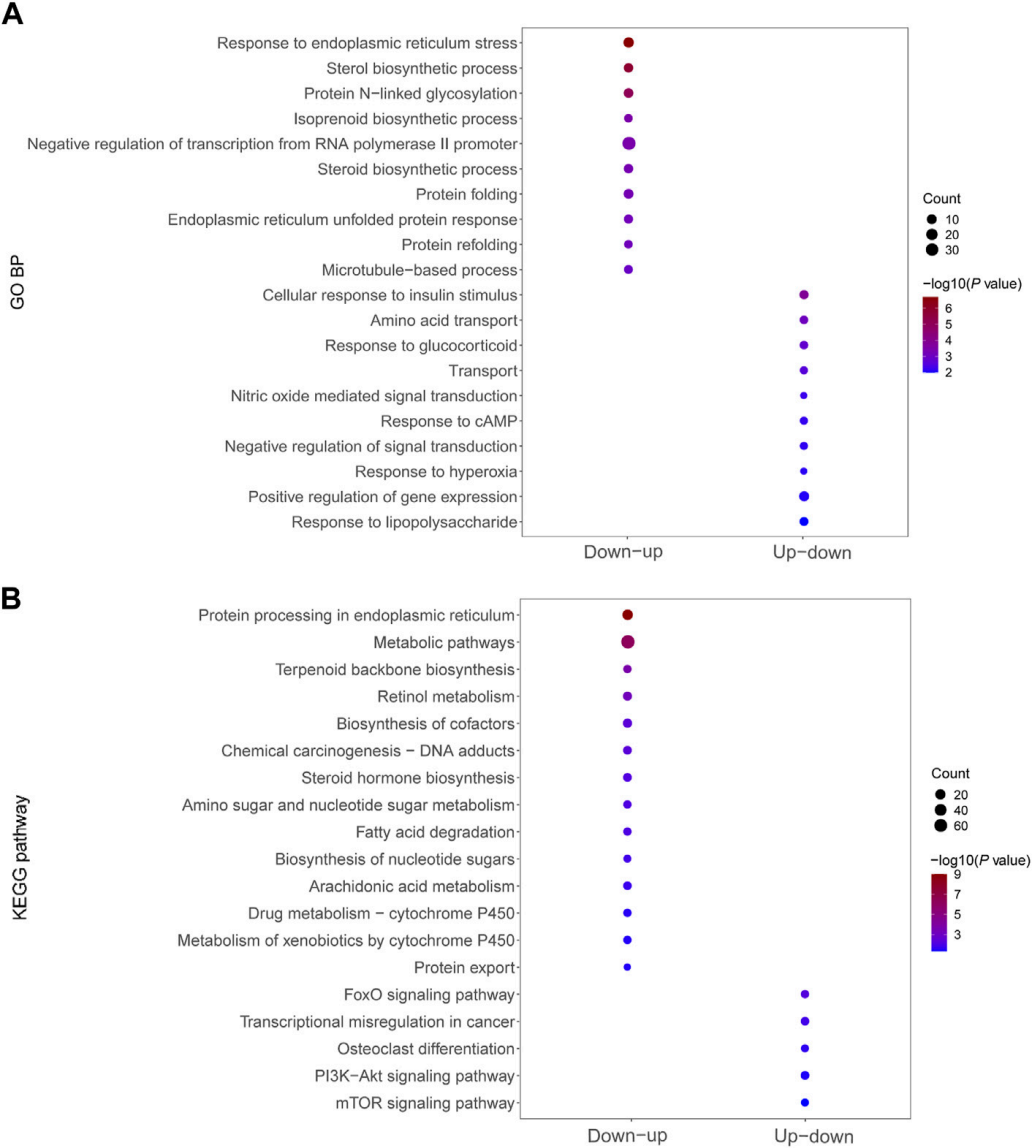
Functional enrichment of dysregulated mRNAs in significant mRNA clusters. **(A)** GO analysis of dysregulated mRNAs down–up/up–down in the Fed–Fast–Refed cycle. **(B)** KEGG signaling enrichment analysis of dysregulated mRNAs down–up/up–down in the Fed–Fast–Refed cycle.

We performed GO analysis and KEGG signaling enrichment analysis of dysregulated mRNAs in significant mRNA clusters. These mRNAs were down–up or up–down regulated in the Fed–Fast–Refed cycle. GO analysis was classified into three categories: biological process, cellular component and molecular function. We only focused on biological process. The top 20 biological process terms of significant mRNA clusters are plotted in Figure 5A. The down–up mRNA clusters in the Fed–Fast–Refed cycle were mainly enriched in response to ER stress, sterol biosynthetic process, protein N-linked glycosylation, and isoprenoid biosynthetic process. The up–down mRNA clusters in the Fed–Fast–Refed cycle were mainly enriched in cellular response to insulin stimulus, amino acid transport, and response to glucocorticoid. The details of GO analysis are summarized in Supplementary Tables S5, S6. The top 20 KEGG pathways of significant mRNA clusters are shown in Figure 5B. The down–up mRNA clusters in the Fed–Fast–Refed cycle were mainly mapped to protein processing in the ER, metabolic pathways, terpenoid backbone biosynthesis, and retinol metabolism. The up–down mRNA clusters in the Fed–Fast–Refed cycle were mainly mapped to the FoxO signaling pathway, transcriptional regulation in cancer, osteoclast differentiation, and the PI3K–Akt signaling pathway. The details of KEGG analysis are summarized in Supplementary Tables S7, S8.

### PPI network of dysregulated mRNAs in significant mRNA clusters

**FIGURE 6:**
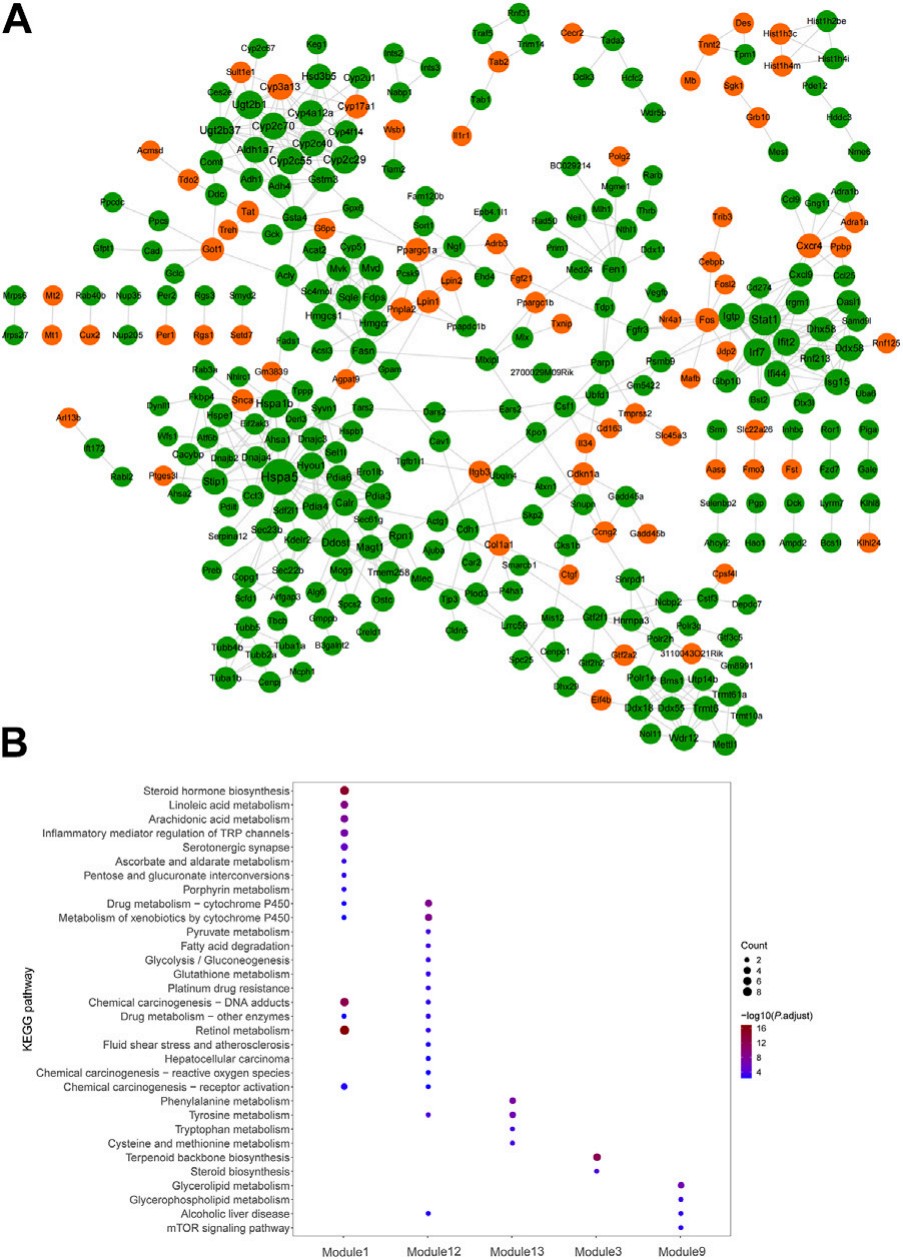
PPI network of dysregulated mRNAs in significant mRNA clusters. **(A)** PPI network predicted the interaction between proteins encoded by down-up/up-down mRNAs in the Fed–Fast–Refed cycle. The orange proteins encoded by dysregulated mRNAs were upregulated and subsequently downregulated in the Fed–Fast–Refed cycle. The green proteins encoded by dysregulated mRNAs were downregulated and subsequently upregulated in the Fed–Fast–Refed cycle. The node size shows the degree of connection. The grey line shows interaction between proteins encoded by these mRNAs. **(B)** Key modules were enriched in a series of KEGG pathways related to metabolism.

Visualized by cytoscape, a PPI was constructed to predict interaction between proteins encoded by down–up and up–down mRNAs in the Fed–Fast–Refed cycle (Figure 6A). Based on the CytoNCA tool, hub genes were identified through ranking Degree Centrality. The top 20 genes encoded Hspa5, Stat1, Ddost, Cyp2c29, Hspa1b, Ugt2b1, Irf7, Ifit2, Cyp2c70, Cyp2c55, Ugt2b37, Igtp, Pdia4, Hyou1, Cyp4a12a, Cyp3a13, Cyp2c40, Aldh1a7, and Fasn. These genes might be pivotal for the Fed–Fast–Refed cycle. Up to 22 key modules clustered in the PPI network were generated using the MCODE tool (Table 3). KEGG pathway enrichment analysis of these key modules was performed (Supplementary Table S9). Importantly, these key modules were mapped to a series of KEGG pathways related to metabolism. Module 1 was enriched in steroid hormone biosynthesis, and linoleic acid and arachidonic acid metabolism (Figure 6B). Module 12 was enriched in drug metabolism by cytochrome P450, metabolism of xenobiotics by cytochrome P450, and tyrosine metabolism. Module 3 was enriched in terpenoid backbone and steroid biosynthesis. Module 9 was enriched in glycerolipid and glycerophospholipid metabolism and alcoholic liver disease.

**TABLE 3:** 22 key modules clustered in PPI network.

Cluster	Score	Nodes	Edges	Node IDs
1	9.11	10	41	Cyp2c40, Cyp2c70, Cyp2c55, Cyp2c29, Aldh1a7, Cyp4a12a, Hsd3b5, Cyp3a13, Ugt2b37, Ugt2b1
2	8.00	8	28	Irf7, Ifit2, Oasl1, Ifi44, Isg15, Dhx58, Ddx58, Stat1
3	8.00	8	28	Fdps, Hmgcr, Mvd, Mvk, Cyp51, Hmgcs1, Sc4mol, Sqle
4	7.75	9	31	Pdia3, Hspa5, Pdia4, Pdia6, Ddost, Calr, Hyou1, Sdf2l1, Dnajc3
5	7.14	8	25	Ddx18, Bms1, Ddx55, Utp14b, Trmt6, Polr1e, Trmt61a, Wdr12
6	5.00	5	10	Tuba1a, Tubb2a, Tubb4b, Tubb5, Tuba1b
7	4.50	5	9	Mlec, Rpn1, Ostc, Tmem258, Magt1
8	4.00	5	8	Hspa1b, Cct3, Ahsa1, Hspe1, Dnaja4
9	4.00	4	6	Lpin2, Ppapdc1b, Lpin1, Pnpla2
10	4.00	4	6	Hist1h4i, Hist1h3c, Hist1h2be, Hist1h4m
11	4.00	4	6	Arfgap3, Kdelr2, Copg1, Sec22b
12	3.33	4	5	Gsta4, Gstm3, Adh1, Adh4
13	3.33	4	5	Got1, Tdo2, Ddc, Tat
14	3.00	3	3	Igtp, Gbp10, Irgm1
15	3.00	3	3	Syvn1, Derl3, Sel1l
16	3.00	3	3	Adra1a, Adra1b, Gng11
17	3.00	3	3	Fkbp4, Cacybp, Stip1
18	3.00	3	3	Pcsk9, Sort1, Ngf
19	3.00	3	3	Mlx, Ppargc1b, Mlxipl
20	3.00	3	3	Ints2, Ints3, Nabp1
21	3.00	3	3	Hnrnpa3, Snrpd1, Ncbp2
22	3.00	3	3	Tpm1, Tnnt2, Des

### miRNA–mRNA regulatory network

**FIGURE 7:**
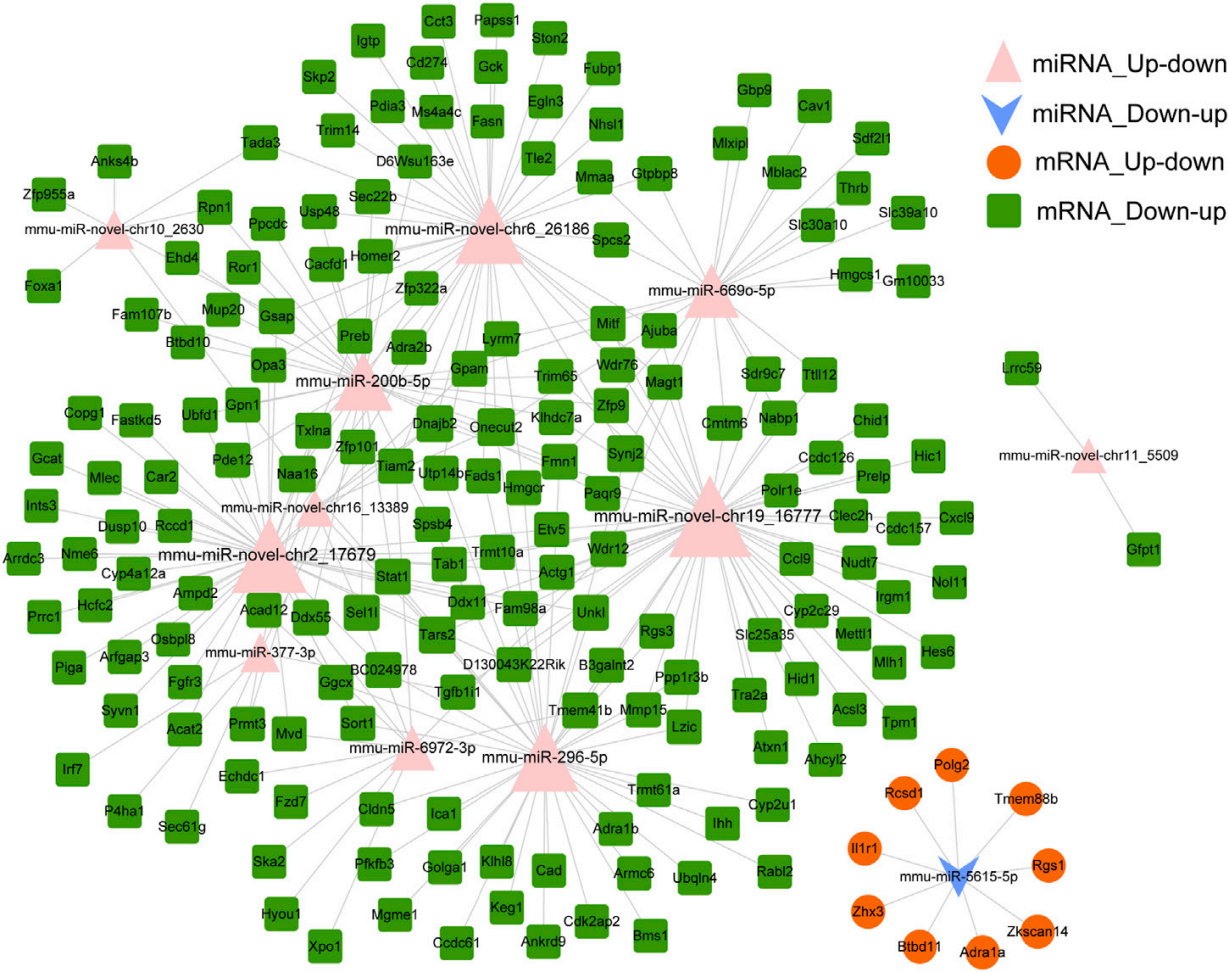
miRNA–mRNA regulatory network. The miRNA–mRNA regulatory network was constructed with 275 miRNA–mRNA pairs in which the regulatory trend between miRNAs and mRNAs was in contrast. The pink triangle indicates miRNAs upregulated and subsequently downregulated in the Fed–Fast–Refed cycle. The blue inverted arrow indicates miRNAs downregulated and subsequently upregulated in the Fed–Fast–Refed cycle. The orange circle indicates mRNAs upregulated and subsequently downregulated in the Fed–Fast–Refed cycle. The green square indicates mRNAs downregulated and subsequently upregulated in the Fed–Fast–Refed cycle. The node size shows the degree of connection. The grey line shows regulatory interaction between miRNA and targeted mRNAs.

miRNAs regulate gene expression after binding with target mRNAs through inhibiting mRNA translation or initiating degradation (Chen et al., 2019). Therefore, potential targets of miRNA were predicted using the miRanda tool to explore the interaction between miRNAs and target mRNAs. In total, 415 miRNA–mRNA pairs were detected based on down–up and up–down mRNAs–miRNAs in the Fed–Fast–Refed cycle. A miRNA–mRNA regulatory network was characterized with 275 miRNA–mRNA pairs (Figure 7). Each up–down miRNA in the Fed–Fast–Refed cycle regulated dozens of down–up mRNAs. Also, down–up miRNAs in the Fed–Fast–Refed cycle regulated dozens of up–down mRNAs. The 415 and 275 miRNA–mRNA pairs are listed in Supplementary Document S5.

### miRNA–mRNA-pathway regulation analysis

**FIGURE 8:**
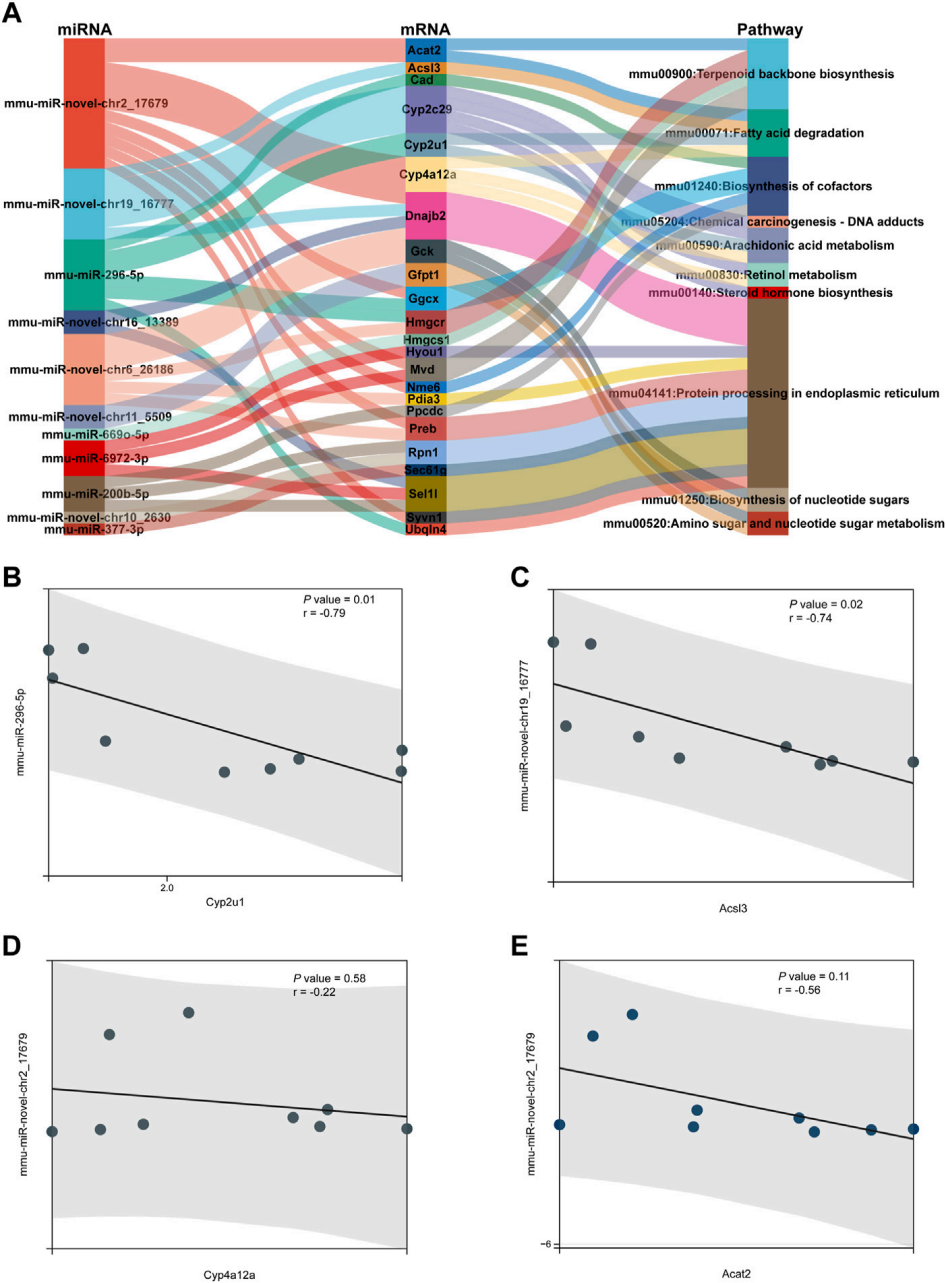
miRNA–mRNA-pathway regulation analysis. **(A)** Sankey diagram was established under joint relation between miRNA–mRNA and mRNA-pathway involved in metabolism. **(B–E)** Scatter plot showing the miRNA–mRNA pairs that participated in lipid metabolism. **(B)** Correlation between mmu-miR-296-5p and Cyp2u1. **(C)** Correlation between mmu-miR-novel-chr19_16777 and Acsl3. **(D)** Correlation between mmu-miR-novel-chr2_17679 and Cyp4a12a. **(E)** Correlation between mmu-miR-novel-chr2_17679 and Acat2.

A Sankey diagram was established between miRNA–mRNA and mRNA pathways involved in metabolism (Figure 8A). Up to 42 miRNA–mRNA-pathway pairs were identified (Table 4). A scatter plot showed the miRNA–mRNA pairs that participated in lipid metabolism (Figures 8B–E). There was a negative correlation between expression of these mRNAs and miRNAs (Figures 8B–E). The correlation between mmu-miR-296-5p and Cyp2u1 and between mmu-miR-novel-chr19_16777 and Acsl3 was significant (*p* < 0.05) (Figures 8B, C).

**TABLE 4:** Identified miRNA–mRNA-pathway pairs.

miRNA	mRNA	KEGG pathway	Type_miRNA	Type_mRNA
mmu-miR-novel-chr2_17679	Acat2	mmu00900:Terpenoid backbone biosynthesis	up-down_mi	down_up_m
mmu-miR-novel-chr2_17679	Acat2	mmu00071:Fatty acid degradation	up-down_mi	down_up_m
mmu-miR-novel-chr19_16777	Acsl3	mmu00071:Fatty acid degradation	up-down_mi	down_up_m
mmu-miR-296-5p	Cad	mmu01240:Biosynthesis of cofactors	up-down_mi	down_up_m
mmu-miR-novel-chr19_16777	Cyp2c29	mmu05204:Chemical carcinogenesis - DNA adducts	up-down_mi	down_up_m
mmu-miR-novel-chr19_16777	Cyp2c29	mmu00590:Arachidonic acid metabolism	up-down_mi	down_up_m
mmu-miR-novel-chr19_16777	Cyp2c29	mmu00830:Retinol metabolism	up-down_mi	down_up_m
mmu-miR-novel-chr19_16777	Cyp2c29	mmu00140:Steroid hormone biosynthesis	up-down_mi	down_up_m
mmu-miR-296-5p	Cyp2u1	mmu00071:Fatty acid degradation	up-down_mi	down_up_m
mmu-miR-296-5p	Cyp2u1	mmu00590:Arachidonic acid metabolism	up-down_mi	down_up_m
mmu-miR-novel-chr2_17679	Cyp4a12a	mmu00590:Arachidonic acid metabolism	up-down_mi	down_up_m
mmu-miR-novel-chr2_17679	Cyp4a12a	mmu00830:Retinol metabolism	up-down_mi	down_up_m
mmu-miR-novel-chr2_17679	Cyp4a12a	mmu00071:Fatty acid degradation	up-down_mi	down_up_m
mmu-miR-novel-chr16_13389	Dnajb2	mmu04141:Protein processing in endoplasmic reticulum	up-down_mi	down_up_m
mmu-miR-novel-chr6_26186	Dnajb2	mmu04141:Protein processing in endoplasmic reticulum	up-down_mi	down_up_m
mmu-miR-novel-chr2_17679	Dnajb2	mmu04141:Protein processing in endoplasmic reticulum	up-down_mi	down_up_m
mmu-miR-novel-chr19_16777	Dnajb2	mmu04141:Protein processing in endoplasmic reticulum	up-down_mi	down_up_m
mmu-miR-novel-chr6_26186	Gck	mmu01250:Biosynthesis of nucleotide sugars	up-down_mi	down_up_m
mmu-miR-novel-chr6_26186	Gck	mmu00520:Amino sugar and nucleotide sugar metabolism	up-down_mi	down_up_m
mmu-miR-novel-chr11_5509	Gfpt1	mmu01250:Biosynthesis of nucleotide sugars	up-down_mi	down_up_m
mmu-miR-novel-chr11_5509	Gfpt1	mmu00520:Amino sugar and nucleotide sugar metabolism	up-down_mi	down_up_m
mmu-miR-novel-chr2_17679	Ggcx	mmu01240:Biosynthesis of cofactors	up-down_mi	down_up_m
mmu-miR-296-5p	Ggcx	mmu01240:Biosynthesis of cofactors	up-down_mi	down_up_m
mmu-miR-novel-chr6_26186	Hmgcr	mmu00900:Terpenoid backbone biosynthesis	up-down_mi	down_up_m
mmu-miR-296-5p	Hmgcr	mmu00900:Terpenoid backbone biosynthesis	up-down_mi	down_up_m
mmu-miR-669o-5p	Hmgcs1	mmu00900:Terpenoid backbone biosynthesis	up-down_mi	down_up_m
mmu-miR-6972-3p	Hyou1	mmu04141:Protein processing in endoplasmic reticulum	up-down_mi	down_up_m
mmu-miR-6972-3p	Mvd	mmu00900:Terpenoid backbone biosynthesis	up-down_mi	down_up_m
mmu-miR-novel-chr2_17679	Mvd	mmu00900:Terpenoid backbone biosynthesis	up-down_mi	down_up_m
mmu-miR-novel-chr2_17679	Nme6	mmu01240:Biosynthesis of cofactors	up-down_mi	down_up_m
mmu-miR-novel-chr6_26186	Pdia3	mmu04141:Protein processing in endoplasmic reticulum	up-down_mi	down_up_m
mmu-miR-200b-5p	Ppcdc	mmu01240:Biosynthesis of cofactors	up-down_mi	down_up_m
mmu-miR-novel-chr2_17679	Preb	mmu04141:Protein processing in endoplasmic reticulum	up-down_mi	down_up_m
mmu-miR-novel-chr6_26186	Preb	mmu04141:Protein processing in endoplasmic reticulum	up-down_mi	down_up_m
mmu-miR-200b-5p	Rpn1	mmu04141:Protein processing in endoplasmic reticulum	up-down_mi	down_up_m
mmu-miR-novel-chr10_2630	Rpn1	mmu04141:Protein processing in endoplasmic reticulum	up-down_mi	down_up_m
mmu-miR-377-3p	Sec61g	mmu04141:Protein processing in endoplasmic reticulum	up-down_mi	down_up_m
mmu-miR-novel-chr16_13389	Sel1l	mmu04141:Protein processing in endoplasmic reticulum	up-down_mi	down_up_m
mmu-miR-200b-5p	Sel1l	mmu04141:Protein processing in endoplasmic reticulum	up-down_mi	down_up_m
mmu-miR-6972-3p	Sel1l	mmu04141:Protein processing in endoplasmic reticulum	up-down_mi	down-up_m
mmu-miR-novel-chr2_17679	Syvn1	mmu04141:Protein processing in endoplasmic reticulum	up-down_mi	down-up_m
mmu-miR-296-5p	Ubqln4	mmu04141:Protein processing in endoplasmic reticulum	up-down_mi	down-up_m

## Discussion

To explore the molecular alterations underlying the physiological Fed–Fast–Refed cycle, we analyzed hepatic mRNA–miRNA expression in mice during the fed to fasted and fasted to refed transitions, based on RNA-Seq and miRNA-Seq. We observed 874 DEGs and 25 dysregulated miRNAs in the liver of mice in the fasted state compared with fed state. A total of 1,048 DEGs and 32 dysregulated miRNAs were captured in the liver of mice in the refed state compared with fasted state. mRNAs in the fasted *versus* fed and refed *versus* fasted cohorts were mainly mapped to 66 and 70 KEGG pathways, respectively. We detected three up–down mRNA clusters, five down–up mRNA clusters, two up–down miRNA clusters and one down–up miRNA cluster during the Fed–Fast–Refed cycle. In addition, we observed up to 22 key modules clustered in a PPI network of proteins encoded by down–up mRNAs and up–down mRNAs in the Fed–Fast–Refed cycle. With 275 miRNA–mRNA pairs in which the regulatory trend between miRNAs and mRNAs was in contrast, a miRNA–mRNA regulatory network was constructed. Up to 42 miRNA–mRNA-pathway pairs were identified between miRNA–mRNA- and mRNA-pathways involved in metabolism.

In the fasted *versus* fed cohort, the most significantly upregulated genes included those encoding Cyp4a14, Cyp4a10, Cyp4a31, and Tnnt2, while the most significantly downregulated genes included those encoding A2m and Serpina12. Serpina12 is an adipokine, that is associated with development of insulin resistance, obesity, and inflammation (Kurowska et al., 2021). Interestingly, Tnnt2, which encodes the cardiac isoform of troponin T, has been shown to regulate hypertrophic cardiomyopathy. Tnnt2-high and Tnnt2-low cardiomyocytes showed differential mitotic activity in response to intracellular glucose (Fajardo et al., 2021). Therefore, we speculate that Tnnt2 expression in the liver could also be regulated by changes in blood glucose levels during fed-fasting-refed cycling. However, its function in the hepatic metabolic regulation remains to be explored in the future studies. In the refed *versus* fasted cohort, the most significantly upregulated genes included those encoding Nrep, Cyp2c69 and Derl3, while the most significantly downregulated genes included those encoding Mt2, Igfbp1, Saa2, Lcn2, and A2m. Igfbp1 is an endogenous promoter of β-cell regeneration and reduces the risk of developing type 2 diabetes (Lu et al., 2016).

GSEA showed that gene expression in the two cohorts shared common pathways, especially for lipid and protein metabolism. In the transition from fed to fasted state and from fasted to refed state, insulin resistance showed a trend to be upregulated. Pathways including fatty acid biosynthesis, steroid biosynthesis, protein export and protein processing in the ER showed a trend to be downregulated during the fed to fasted transition. However, these pathways showed a trend to be upregulated during the fasted to refed transition.

GO analysis demonstrated that down–up mRNA clusters in the Fed–Fast–Refed cycle were mainly related to response to ER stress, sterol biosynthetic process, protein N-linked glycosylation, and isoprenoid biosynthetic process. The up–down mRNA clusters were mainly related to cellular response to insulin stimulus, amino acid transport, and response to glucocorticoid. KEGG pathway analysis found that down–up mRNA clusters in the Fed–Fast–Refed cycle were mainly mapped to protein processing in the ER, metabolic pathways, terpenoid backbone biosynthesis, and retinol metabolism. Up–down mRNA clusters in the Fed–Fast–Refed cycle were mainly mapped to the FoxO signaling pathway, transcriptional misregulation in cancer, osteoclast differentiation, and the PI3K–Akt signaling pathway.

Hub genes were identified through PPI network analysis of proteins encoded by down–up and up–down mRNAs in the Fed–Fast–Refed cycle. We found that the top 20 hub genes included those encoding Hspa5, Stat1, Ddost, Cyp2c29, Hspa1b, Ugt2b1, Irf7, Ifit2, Cyp2c70, Cyp2c55, Ugt2b37, Igtp, Pdia4, Hyou1, Cyp4a12a, Cyp3a13, Cyp2c40, Aldh1a7, and Fasn. These hub genes included several classes with distinct functions. For instance, Cyp2c29, Cyp2c70, Cyp2c55, Cyp4a12a, Cyp3a13, and Cyp2c40 are cytochrome P450 (CYP) enzymes are involved in the metabolism of drugs, steroids and carcinogens (Guengerich et al., 2016; Zhao et al., 2021). Hspa5 and Hspa1b encode proteins which localized in the ER lumen. Overexpression of Hspa5 on the cell membrane mediates the vast number of disordered proteins produced under stress (Wang et al., 2017). Ugt2b1 and Ugt2b37 belong to UDP glucuronosyltransferase 2 family. UDP glucuronosyltransferase prevents the accumulation of potentially toxic compounds and their subsequent bioactivation to more toxic intermediates (Grancharov et al., 2001; Rowland et al., 2013). Functional enrichment analysis confirmed that steroid biosynthesis, and drug metabolism by cytochrome P450 or other enzymes were enriched by key modules in the PPI network. Importantly, these key modules were also mapped to a series of KEGG pathways related to lipid metabolism, including steroid biosynthesis, and linoleic acid, arachidonic acid, glycerolipid and glycerophospholipid metabolism.

Through analysis of miRNA–mRNA-pathway regulation, we found that several miRNA–mRNA pairs participated in lipid metabolism. The target mRNA expression was negatively regulated by miRNA expression. There was a significant correlation between mmu-miR-296-5p and Cyp2u1 as well as between mmu-miR-novel-chr19_16777 and Acsl3. Cyp2u1 has been reported to mediate hydroxylation of arachidonic acid metabolism (Yu et al., 2019). ACSL3 is regarded as a novel GABARAPL2 interactor that links ufmylation and lipid droplet biogenesis (Eck et al., 2020).

There were several limitations to our study. First, only three replicates were involved in each group. Second, our findings were based on murine models. Third, to further explore the role and mechanisms of significant genes and miRNAs in the physiological Fed–Fast–Refed cycle, functional experiments should be carried out.

In conclusion, this study identified several novel potential mRNAs and miRNAs through expression trend analysis and regulation networks. These up–down and down–up mRNAs and miRNAs might be involved in lipid metabolism during the physiological Fed–Fast–Refed cycle.

## Data Availability

The datasets presented in this study can be found in online repositories. The names of the repository/repositories and accession number (s) can be found below: https://www.ncbi.nlm.nih.gov/geo/query/acc.cgi?acc=GSE225697.
