# Supplementary material for: Hepatic gene expression profiles during fed–fasted–refed state in mice
Source: Front Genet. 2023 Mar 3;14:1145769. doi: 10.3389/fgene.2023.1145769 (PMC10020372; doi:10.3389/fgene.2023.1145769)
Supplement: Supplementary file 1 [file Table1.DOCX]

**Supplementary Information**

**Supplementary Figure**

**Supplementary Figure 1. Construction and validation of mice during fed-fasted-refed states.** (A) Blood glucose levels among three groups of mice. (B-C) Relative mRNA expression levels of gluconeogenic (B) and (C) lipogenic genes in the livers. n=5 per group.


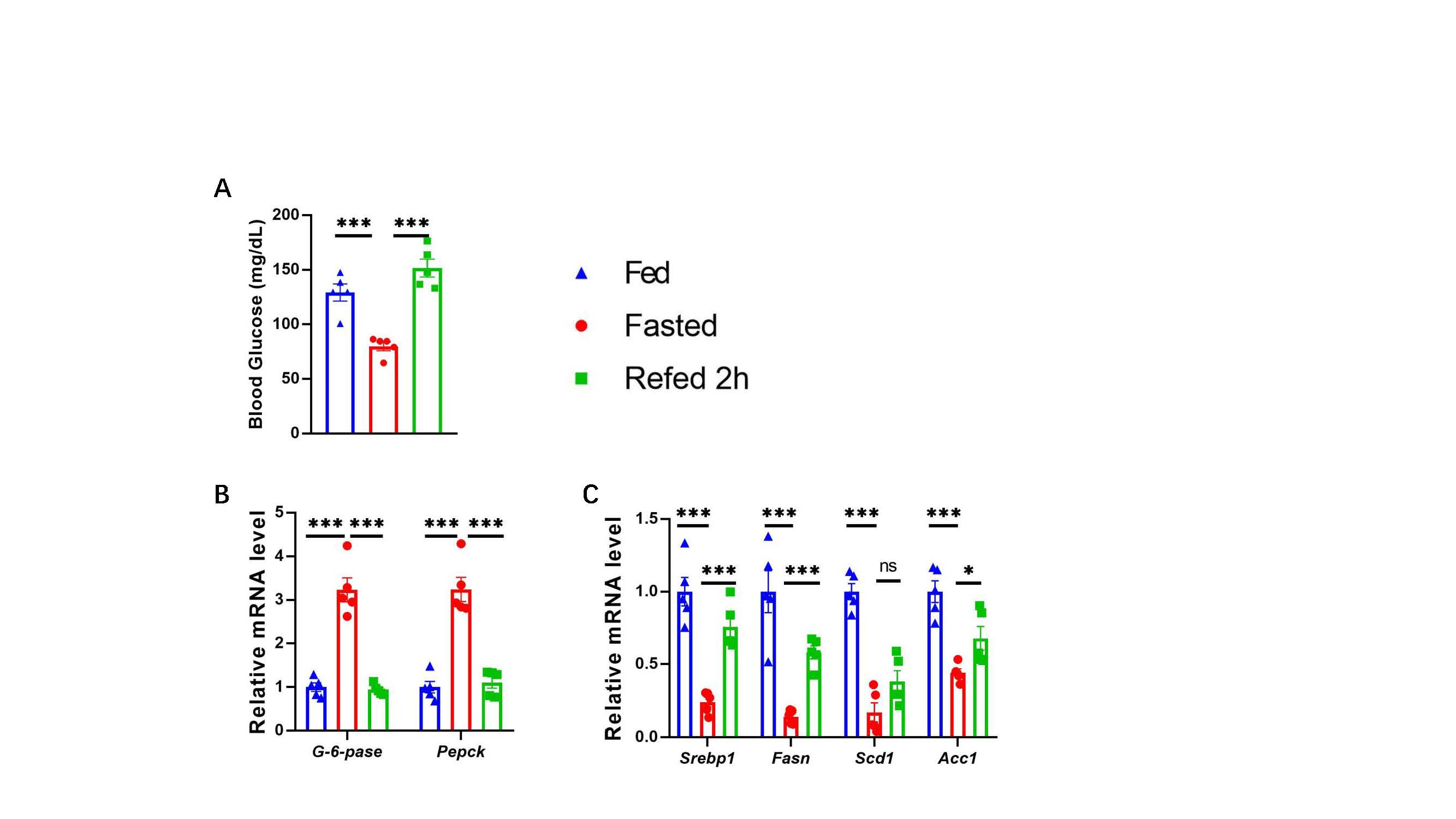


**
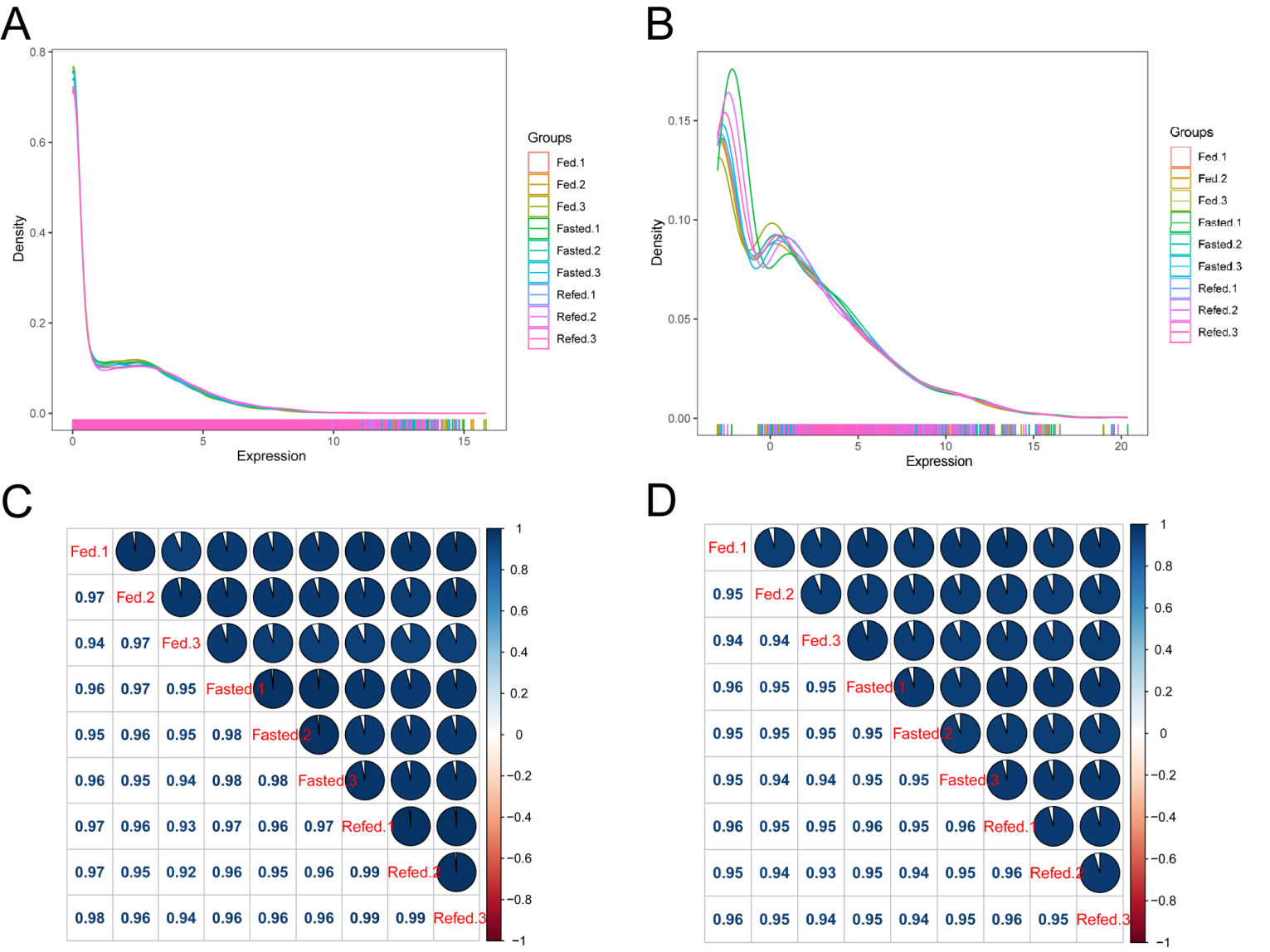
**

**Supplementary Figure 2. Expression density and spearman correlation analysis of mRNA-Seq and miRNA-Seq datasets**

1. B) mRNA (A) and miRNA (B) expression density in fed, fasted and refed groups. (C-D) Spearman correlation analysis of mRNA-Seq (C) and miRNA-Seq (D) profile in in fed, fasted and refed groups.

**Supplementary Table**

**Table S1. Up-regulated KEGG pathways in fasted versus fed cohort based on GSEA.**

| ID | Description | enrichmentScore | NES | *P* value | *P*.adjust | rank |
| --- | --- | --- | --- | --- | --- | --- |
| mmu03320 | PPAR signaling pathway | 0.533624 | 2.062431 | 0.000019 | 0.006072 | 1163 |
| mmu00220 | Arginine biosynthesis | 0.743585 | 2.120406 | 0.000144 | 0.009247 | 1006 |
| mmu00410 | beta-Alanine metabolism | 0.676578 | 2.096367 | 0.000185 | 0.009877 | 1604 |
| mmu00280 | Valine, leucine and isoleucine degradation | 0.522192 | 1.886303 | 0.000484 | 0.014488 | 1608 |
| mmu00270 | Cysteine and methionine metabolism | 0.535386 | 1.879010 | 0.000496 | 0.014488 | 272 |
| mmu00310 | Lysine degradation | 0.485827 | 1.822059 | 0.000628 | 0.015948 | 2106 |
| mmu01230 | Biosynthesis of amino acids | 0.439772 | 1.703881 | 0.000838 | 0.016804 | 694 |
| mmu04146 | Peroxisome | 0.423571 | 1.717510 | 0.000931 | 0.017572 | 1427 |
| mmu00250 | Alanine, aspartate and glutamate metabolism | 0.567196 | 1.842222 | 0.001704 | 0.026046 | 646 |
| mmu05017 | Spinocerebellar ataxia | 0.363941 | 1.519114 | 0.001810 | 0.026408 | 3197 |
| mmu00071 | Fatty acid degradation | 0.480755 | 1.700284 | 0.004031 | 0.047922 | 1604 |
| mmu00360 | Phenylalanine metabolism | 0.728391 | 1.827863 | 0.005014 | 0.057484 | 785 |
| mmu00430 | Taurine and hypotaurine metabolism | 0.719309 | 1.748797 | 0.007437 | 0.079572 | 583 |
| mmu04979 | Cholesterol metabolism | 0.439504 | 1.587612 | 0.009074 | 0.093399 | 2547 |
| mmu00120 | Primary bile acid biosynthesis | 0.631522 | 1.762195 | 0.009311 | 0.093399 | 567 |
| mmu04931 | Insulin resistance | 0.357967 | 1.461820 | 0.010802 | 0.101984 | 1051 |
| mmu00770 | Pantothenate and CoA biosynthesis | 0.577392 | 1.720787 | 0.012912 | 0.118426 | 1109 |
| mmu00380 | Tryptophan metabolism | 0.446712 | 1.526707 | 0.017228 | 0.141801 | 1604 |
| mmu00640 | Propanoate metabolism | 0.481634 | 1.564323 | 0.019956 | 0.155796 | 1608 |
| mmu00260 | Glycine, serine and threonine metabolism | 0.439415 | 1.501767 | 0.020384 | 0.155796 | 1107 |
| mmu01210 | 2-Oxocarboxylic acid metabolism | 0.578788 | 1.615046 | 0.022483 | 0.160381 | 597 |
| mmu04975 | Fat digestion and absorption | 0.485637 | 1.518480 | 0.030929 | 0.201359 | 840 |
| mmu00340 | Histidine metabolism | 0.557409 | 1.555391 | 0.034250 | 0.211428 | 1758 |
| mmu00130 | Ubiquinone and other terpenoid-quinone biosynthesis | 0.624906 | 1.568174 | 0.039106 | 0.223312 | 179 |
| mmu05144 | Malaria | 0.459887 | 1.469945 | 0.041045 | 0.223312 | 500 |
| mmu05415 | Diabetic cardiomyopathy | 0.278256 | 1.216266 | 0.042203 | 0.225785 | 2761 |
| mmu00564 | Glycerophospholipid metabolism | 0.365442 | 1.415876 | 0.045637 | 0.232533 | 543 |
| mmu00330 | Arginine and proline metabolism | 0.400923 | 1.384996 | 0.047602 | 0.238680 | 1604 |
| mmu04913 | Ovarian steroidogenesis | 0.445496 | 1.423944 | 0.048331 | 0.238680 | 645 |

**Table S2. Down-regulated KEGG pathways in fasted versus fed cohort based on GSEA.**

| ID | Description | enrichmentScore | NES | *P* value | *P*.adjust | rank |
| --- | --- | --- | --- | --- | --- | --- |
| mmu05160 | Hepatitis C | -0.502202 | -1.791908 | 0.000043 | 0.006223 | 2012 |
| mmu03030 | DNA replication | -0.681991 | -1.968075 | 0.000058 | 0.006223 | 1758 |
| mmu04141 | Protein processing in endoplasmic reticulum | -0.459472 | -1.676465 | 0.000122 | 0.009247 | 2716 |
| mmu05169 | Epstein-Barr virus infection | -0.439646 | -1.618062 | 0.000383 | 0.014488 | 2012 |
| mmu04110 | Cell cycle | -0.482317 | -1.688538 | 0.000438 | 0.014488 | 919 |
| mmu01250 | Biosynthesis of nucleotide sugars | -0.624790 | -1.815508 | 0.000446 | 0.014488 | 716 |
| mmu00100 | Steroid biosynthesis | -0.762462 | -1.805434 | 0.000686 | 0.015948 | 728 |
| mmu05162 | Measles | -0.480124 | -1.673290 | 0.000696 | 0.015948 | 2012 |
| mmu04514 | Cell adhesion molecules | -0.520983 | -1.702294 | 0.000836 | 0.016804 | 2246 |
| mmu00520 | Amino sugar and nucleotide sugar metabolism | -0.569397 | -1.736305 | 0.001135 | 0.020246 | 1055 |
| mmu00510 | N-Glycan biosynthesis | -0.571897 | -1.755874 | 0.001215 | 0.020534 | 3024 |
| mmu04060 | Cytokine-cytokine receptor interaction | -0.454707 | -1.599049 | 0.001448 | 0.023247 | 1936 |
| mmu05215 | Prostate cancer | -0.493692 | -1.670812 | 0.001956 | 0.027295 | 1838 |
| mmu04657 | IL-17 signaling pathway | -0.527417 | -1.670191 | 0.002375 | 0.031765 | 1040 |
| mmu04540 | Gap junction | -0.518214 | -1.630509 | 0.003146 | 0.040399 | 1633 |
| mmu05164 | Influenza A | -0.436372 | -1.554564 | 0.003920 | 0.047922 | 1898 |
| mmu05203 | Viral carcinogenesis | -0.397307 | -1.449359 | 0.005671 | 0.062771 | 2841 |
| mmu00970 | Aminoacyl-tRNA biosynthesis | -0.515458 | -1.571826 | 0.010014 | 0.097414 | 4319 |
| mmu03440 | Homologous recombination | -0.577206 | -1.596499 | 0.013825 | 0.123271 | 1528 |
| mmu04145 | Phagosome | -0.405355 | -1.441842 | 0.014838 | 0.128728 | 2246 |
| mmu05416 | Viral myocarditis | -0.507866 | -1.549290 | 0.016094 | 0.135956 | 2246 |
| mmu05222 | Small cell lung cancer | -0.440441 | -1.470022 | 0.019935 | 0.155796 | 2333 |
| mmu00513 | Various types of N-glycan biosynthesis | -0.527462 | -1.546166 | 0.021149 | 0.157881 | 2454 |
| mmu05166 | Human T-cell leukemia virus 1 infection | -0.362028 | -1.335951 | 0.021767 | 0.158803 | 2668 |
| mmu04974 | Protein digestion and absorption | -0.529926 | -1.529248 | 0.028164 | 0.195243 | 1235 |
| mmu05219 | Bladder cancer | -0.529352 | -1.527594 | 0.028587 | 0.195243 | 601 |
| mmu03430 | Mismatch repair | -0.588746 | -1.528423 | 0.030567 | 0.201359 | 2850 |
| mmu04622 | RIG-I-like receptor signaling pathway | -0.483105 | -1.483259 | 0.031364 | 0.201359 | 977 |
| mmu03060 | Protein export | -0.545855 | -1.495638 | 0.034160 | 0.211428 | 1071 |
| mmu05168 | Herpes simplex virus 1 infection | -0.332932 | -1.275165 | 0.036765 | 0.222669 | 2269 |
| mmu00061 | Fatty acid biosynthesis | -0.653428 | -1.521625 | 0.038760 | 0.223312 | 865 |
| mmu03410 | Base excision repair | -0.515940 | -1.448103 | 0.040115 | 0.223312 | 1268 |
| mmu00052 | Galactose metabolism | -0.520002 | -1.442418 | 0.040346 | 0.223312 | 2040 |
| mmu05161 | Hepatitis B | -0.372401 | -1.337514 | 0.040509 | 0.223312 | 2277 |
| mmu04610 | Complement and coagulation cascades | -0.419208 | -1.396329 | 0.043810 | 0.230108 | 3119 |
| mmu04530 | Tight junction | -0.376283 | -1.342616 | 0.044444 | 0.230108 | 2841 |
| mmu05132 | Salmonella infection | -0.337679 | -1.265609 | 0.049288 | 0.239719 | 2841 |

**Table S3. Up-regulated KEGG pathways in refed versus fasted cohort based on GSEA.**

| ID | Description | Enrichment Score | NES | *P* value | *P*.adjust | rank |
| --- | --- | --- | --- | --- | --- | --- |
| mmu04141 | Protein processing in endoplasmic reticulum | 0.600713 | 2.309850 | 0.000000 | 0.000000 | 1335 |
| mmu03040 | Spliceosome | 0.456839 | 1.707235 | 0.000094 | 0.004965 | 3208 |
| mmu00980 | Metabolism of xenobiotics by cytochrome P450 | 0.582824 | 1.868450 | 0.000104 | 0.004965 | 2407 |
| mmu01240 | Biosynthesis of cofactors | 0.455723 | 1.715914 | 0.000106 | 0.004965 | 3435 |
| mmu00982 | Drug metabolism - cytochrome P450 | 0.562338 | 1.821225 | 0.000224 | 0.007989 | 2407 |
| mmu03060 | Protein export | 0.672873 | 1.879226 | 0.000256 | 0.008185 | 2137 |
| mmu00970 | Aminoacyl-tRNA biosynthesis | 0.584149 | 1.831316 | 0.000303 | 0.008185 | 2685 |
| mmu05168 | Herpes simplex virus 1 infection | 0.370797 | 1.537130 | 0.000306 | 0.008185 | 2631 |
| mmu00900 | Terpenoid backbone biosynthesis | 0.668886 | 1.826121 | 0.000510 | 0.012592 | 850 |
| mmu00830 | Retinol metabolism | 0.517382 | 1.736711 | 0.000674 | 0.015445 | 1092 |
| mmu00071 | Fatty acid degradation | 0.542863 | 1.716098 | 0.001026 | 0.018584 | 2066 |
| mmu05204 | Chemical carcinogenesis - DNA adducts | 0.519706 | 1.710937 | 0.001042 | 0.018584 | 2407 |
| mmu01250 | Biosynthesis of nucleotide sugars | 0.578188 | 1.720280 | 0.001631 | 0.027294 | 1360 |
| mmu04913 | Ovarian steroidogenesis | 0.609293 | 1.710146 | 0.001730 | 0.027294 | 365 |
| mmu04950 | Maturity onset diabetes of the young | 0.713544 | 1.734781 | 0.002075 | 0.028474 | 1007 |
| mmu03008 | Ribosome biogenesis in eukaryotes | 0.465417 | 1.592870 | 0.002129 | 0.028474 | 3243 |
| mmu03013 | Nucleocytoplasmic transport | 0.427113 | 1.542462 | 0.002370 | 0.029266 | 4182 |
| mmu04612 | Antigen processing and presentation | 0.499743 | 1.613218 | 0.003636 | 0.040248 | 767 |
| mmu00983 | Drug metabolism - other enzymes | 0.444125 | 1.552585 | 0.004624 | 0.049472 | 2149 |
| mmu00140 | Steroid hormone biosynthesis | 0.470854 | 1.562142 | 0.006219 | 0.059516 | 1399 |
| mmu05012 | Parkinson disease | 0.354453 | 1.421060 | 0.006509 | 0.059695 | 5065 |
| mmu00062 | Fatty acid elongation | 0.607955 | 1.651342 | 0.007961 | 0.063117 | 1979 |
| mmu00520 | Amino sugar and nucleotide sugar metabolism | 0.503986 | 1.580005 | 0.008364 | 0.063926 | 1360 |
| mmu00860 | Porphyrin metabolism | 0.517003 | 1.589853 | 0.012336 | 0.090237 | 2407 |
| mmu00100 | Steroid biosynthesis | 0.613121 | 1.564039 | 0.013415 | 0.091624 | 982 |
| mmu00190 | Oxidative phosphorylation | 0.394012 | 1.436118 | 0.016070 | 0.105272 | 4847 |
| mmu05014 | Amyotrophic lateral sclerosis | 0.324772 | 1.346332 | 0.018815 | 0.119754 | 5065 |
| mmu05020 | Prion disease | 0.333160 | 1.334506 | 0.027062 | 0.155125 | 5065 |
| mmu00650 | Butanoate metabolism | 0.561021 | 1.523858 | 0.028062 | 0.158033 | 1895 |
| mmu04918 | Thyroid hormone synthesis | 0.476213 | 1.480878 | 0.034574 | 0.181941 | 2593 |
| mmu00053 | Ascorbate and aldarate metabolism | 0.520610 | 1.461235 | 0.038943 | 0.189404 | 2595 |
| mmu00061 | Fatty acid biosynthesis | 0.607746 | 1.477565 | 0.045911 | 0.213586 | 3743 |
| mmu00040 | Pentose and glucuronate interconversions | 0.493966 | 1.433578 | 0.048011 | 0.220165 | 2407 |

**Table S4. Down-regulated KEGG pathways in refed versus fasted cohort based on GSEA.**

| ID | Description | Enrichment Score | NES | *P* value | *P*.adjust | rank |
| --- | --- | --- | --- | --- | --- | --- |
| mmu04610 | Complement and coagulation cascades | -0.508563 | -2.078135 | 0.000011 | 0.001814 | 1647 |
| mmu04115 | p53 signaling pathway | -0.527071 | -2.015734 | 0.000077 | 0.004965 | 501 |
| mmu04060 | Cytokine-cytokine receptor interaction | -0.437426 | -1.840915 | 0.000108 | 0.004965 | 941 |
| mmu04613 | Neutrophil extracellular trap formation | -0.446242 | -1.821978 | 0.000190 | 0.007606 | 903 |
| mmu04512 | ECM-receptor interaction | -0.553083 | -1.896079 | 0.000852 | 0.017505 | 992 |
| mmu05146 | Amoebiasis | -0.489318 | -1.885792 | 0.000873 | 0.017505 | 1033 |
| mmu04151 | PI3K-Akt signaling pathway | -0.309203 | -1.483397 | 0.001786 | 0.027294 | 1220 |
| mmu00220 | Arginine biosynthesis | -0.655856 | -1.874149 | 0.002105 | 0.028474 | 1085 |
| mmu05202 | Transcriptional misregulation in cancer | -0.366534 | -1.569269 | 0.002304 | 0.029266 | 598 |
| mmu05150 | Staphylococcus aureus infection | -0.524619 | -1.809138 | 0.003100 | 0.035977 | 1647 |
| mmu04978 | Mineral absorption | -0.572910 | -1.779245 | 0.003138 | 0.035977 | 916 |
| mmu04068 | FoxO signaling pathway | -0.365739 | -1.533008 | 0.004958 | 0.049732 | 1213 |
| mmu05205 | Proteoglycans in cancer | -0.326393 | -1.468367 | 0.004958 | 0.049732 | 1348 |
| mmu04380 | Osteoclast differentiation | -0.388972 | -1.589978 | 0.006304 | 0.059516 | 1287 |
| mmu04150 | mTOR signaling pathway | -0.332341 | -1.456295 | 0.007433 | 0.063117 | 1276 |
| mmu04061 | Viral protein interaction with cytokine and cytokine receptor | -0.512462 | -1.756186 | 0.007793 | 0.063117 | 655 |
| mmu05206 | MicroRNAs in cancer | -0.345996 | -1.479466 | 0.007955 | 0.063117 | 1328 |
| mmu04012 | ErbB signaling pathway | -0.420334 | -1.627638 | 0.008027 | 0.063117 | 1357 |
| mmu04080 | Neuroactive ligand-receptor interaction | -0.409120 | -1.569498 | 0.008062 | 0.063117 | 718 |
| mmu05410 | Hypertrophic cardiomyopathy | -0.497722 | -1.705671 | 0.012369 | 0.090237 | 400 |
| mmu05414 | Dilated cardiomyopathy | -0.497059 | -1.703401 | 0.012798 | 0.091291 | 848 |
| mmu04010 | MAPK signaling pathway | -0.290726 | -1.353640 | 0.013337 | 0.091624 | 1220 |
| mmu04640 | Hematopoietic cell lineage | -0.448041 | -1.627182 | 0.013703 | 0.091639 | 931 |
| mmu04064 | NF-kappa B signaling pathway | -0.372920 | -1.490380 | 0.019082 | 0.119754 | 903 |
| mmu05322 | Systemic lupus erythematosus | -0.479183 | -1.642140 | 0.019399 | 0.119754 | 1640 |
| mmu05210 | Colorectal cancer | -0.352835 | -1.445997 | 0.020585 | 0.124677 | 1455 |
| mmu04936 | Alcoholic liver disease | -0.316927 | -1.350380 | 0.020981 | 0.124720 | 1291 |
| mmu04142 | Lysosome | -0.312415 | -1.349978 | 0.021557 | 0.125815 | 1616 |
| mmu04931 | Insulin resistance | -0.343187 | -1.412101 | 0.029541 | 0.163493 | 1197 |
| mmu04920 | Adipocytokine signaling pathway | -0.375050 | -1.409993 | 0.030773 | 0.167428 | 655 |
| mmu04630 | JAK-STAT signaling pathway | -0.319984 | -1.317126 | 0.031479 | 0.168412 | 1357 |
| mmu04611 | Platelet activation | -0.343891 | -1.401196 | 0.035305 | 0.182788 | 1127 |
| mmu04974 | Protein digestion and absorption | -0.447962 | -1.472854 | 0.035895 | 0.182893 | 1224 |
| mmu04657 | IL-17 signaling pathway | -0.377172 | -1.447654 | 0.037557 | 0.188373 | 530 |
| mmu04662 | B cell receptor signaling pathway | -0.355424 | -1.359287 | 0.038703 | 0.189404 | 1287 |
| mmu04152 | AMPK signaling pathway | -0.299438 | -1.252288 | 0.044739 | 0.211567 | 1211 |
| mmu05221 | Acute myeloid leukemia | -0.356243 | -1.366647 | 0.044818 | 0.211567 | 1357 |

**Table S5. GO analysis of down-up mRNAs in Fed-Fasted-Refed cycle.**

| GO BP term | Count | *P* value |
| --- | --- | --- |
| GO:0034976~response to endoplasmic reticulum stress | 13 | 0.000000 |
| GO:0016126~sterol biosynthetic process | 8 | 0.000002 |
| GO:0006487~protein N-linked glycosylation | 9 | 0.000009 |
| GO:0008299~isoprenoid biosynthetic process | 5 | 0.000317 |
| GO:0000122~negative regulation of transcription from RNA polymerase II promoter | 37 | 0.000380 |
| GO:0006694~steroid biosynthetic process | 8 | 0.000479 |
| GO:0006457~protein folding | 10 | 0.000514 |
| GO:0030968~endoplasmic reticulum unfolded protein response | 7 | 0.000675 |
| GO:0042026~protein refolding | 5 | 0.000748 |
| GO:0007017~microtubule-based process | 6 | 0.001075 |
| GO:0006986~response to unfolded protein | 7 | 0.001197 |
| GO:0030433~ubiquitin-dependent ERAD pathway | 8 | 0.001257 |
| GO:0006695~cholesterol biosynthetic process | 6 | 0.001353 |
| GO:0071346~cellular response to interferon-gamma | 9 | 0.001669 |
| GO:0000278~mitotic cell cycle | 10 | 0.002199 |
| GO:0006888~ER to Golgi vesicle-mediated transport | 9 | 0.002216 |
| GO:0008202~steroid metabolic process | 10 | 0.002308 |
| GO:0051607~defense response to virus | 13 | 0.003314 |
| GO:0006082~organic acid metabolic process | 6 | 0.003598 |
| GO:0042738~exogenous drug catabolic process | 6 | 0.003916 |
| GO:0009615~response to virus | 8 | 0.003953 |
| GO:0018279~protein N-linked glycosylation via asparagine | 4 | 0.004510 |
| GO:0097267~omega-hydroxylase P450 pathway | 3 | 0.005789 |
| GO:0031398~positive regulation of protein ubiquitination | 7 | 0.005954 |
| GO:0061077~chaperone-mediated protein folding | 5 | 0.006406 |
| GO:0019373~epoxygenase P450 pathway | 5 | 0.006406 |
| GO:0016567~protein ubiquitination | 18 | 0.006478 |
| GO:0006974~cellular response to DNA damage stimulus | 20 | 0.007942 |
| GO:0008203~cholesterol metabolic process | 8 | 0.009269 |
| GO:0008152~metabolic process | 10 | 0.011513 |
| GO:0006281~DNA repair | 16 | 0.011853 |
| GO:0006805~xenobiotic metabolic process | 7 | 0.012623 |
| GO:0009306~protein secretion | 5 | 0.012742 |
| GO:0006629~lipid metabolic process | 25 | 0.013356 |
| GO:1901998~toxin transport | 5 | 0.013738 |
| GO:0019369~arachidonic acid metabolic process | 5 | 0.014783 |
| GO:0009617~response to bacterium | 13 | 0.015303 |
| GO:0006084~acetyl-CoA metabolic process | 3 | 0.016462 |
| GO:1901838~positive regulation of transcription of nuclear large rRNA transcript from RNA polymerase I promoter | 3 | 0.016462 |
| GO:0071353~cellular response to interleukin-4 | 4 | 0.016719 |
| GO:0032781~positive regulation of ATPase activity | 4 | 0.016719 |
| GO:0032728~positive regulation of interferon-beta production | 5 | 0.017019 |
| GO:0045721~negative regulation of gluconeogenesis | 4 | 0.018450 |
| GO:0030488~tRNA methylation | 4 | 0.018450 |
| GO:0031017~exocrine pancreas development | 3 | 0.019854 |
| GO:0043039~tRNA aminoacylation | 3 | 0.027418 |
| GO:0031397~negative regulation of protein ubiquitination | 5 | 0.028010 |
| GO:0006284~base-excision repair | 4 | 0.028545 |
| GO:0051085~chaperone mediated protein folding requiring cofactor | 4 | 0.028545 |
| GO:0060348~bone development | 6 | 0.030216 |
| GO:0090305~nucleic acid phosphodiester bond hydrolysis | 3 | 0.031567 |
| GO:1902902~negative regulation of autophagosome assembly | 3 | 0.031567 |
| GO:0033148~positive regulation of intracellular estrogen receptor signaling pathway | 3 | 0.031567 |
| GO:0031016~pancreas development | 4 | 0.033252 |
| GO:0006730~one-carbon metabolic process | 4 | 0.035747 |
| GO:0032092~positive regulation of protein binding | 6 | 0.038752 |
| GO:0043410~positive regulation of MAPK cascade | 9 | 0.039348 |
| GO:1903892~negative regulation of ATF6-mediated unfolded protein response | 2 | 0.040003 |
| GO:0046785~microtubule polymerization | 3 | 0.040541 |
| GO:0006641~triglyceride metabolic process | 4 | 0.043792 |
| GO:0009408~response to heat | 5 | 0.044460 |
| GO:0033574~response to testosterone | 4 | 0.046657 |
| GO:0006511~ubiquitin-dependent protein catabolic process | 11 | 0.048489 |

**Table S6. GO analysis of of up-down mRNAs in Fed-Fasted-Refed cycle.**

| GO BP term | Count | *P* value |
| --- | --- | --- |
| GO:0032869~cellular response to insulin stimulus | 7 | 0.000160 |
| GO:0006865~amino acid transport | 5 | 0.000805 |
| GO:0051384~response to glucocorticoid | 5 | 0.001610 |
| GO:0006810~transport | 4 | 0.002621 |
| GO:0007263~nitric oxide mediated signal transduction | 3 | 0.005577 |
| GO:0051591~response to cAMP | 4 | 0.006253 |
| GO:0009968~negative regulation of signal transduction | 4 | 0.006932 |
| GO:0055093~response to hyperoxia | 3 | 0.007837 |
| GO:0010628~positive regulation of gene expression | 11 | 0.008534 |
| GO:0032496~response to lipopolysaccharide | 6 | 0.010652 |
| GO:0007568~aging | 6 | 0.011584 |
| GO:0045944~positive regulation of transcription from RNA polymerase II promoter | 17 | 0.012097 |
| GO:0045893~positive regulation of transcription, DNA-templated | 12 | 0.012349 |
| GO:0007585~respiratory gaseous exchange | 3 | 0.012362 |
| GO:0071280~cellular response to copper ion | 3 | 0.012362 |
| GO:0006520~cellular amino acid metabolic process | 3 | 0.013375 |
| GO:0043373~CD4-positive, alpha-beta T cell lineage commitment | 2 | 0.014084 |
| GO:0044344~cellular response to fibroblast growth factor stimulus | 3 | 0.018953 |
| GO:1901215~negative regulation of neuron death | 4 | 0.020393 |
| GO:0014048~regulation of glutamate secretion | 2 | 0.021052 |
| GO:1904640~response to methionine | 2 | 0.021052 |
| GO:0045672~positive regulation of osteoclast differentiation | 3 | 0.021414 |
| GO:0071276~cellular response to cadmium ion | 3 | 0.024000 |
| GO:0006366~transcription from RNA polymerase II promoter | 6 | 0.026700 |
| GO:0042493~response to drug | 7 | 0.027760 |
| GO:0006844~acyl carnitine transport | 2 | 0.027971 |
| GO:0009058~biosynthetic process | 3 | 0.029530 |
| GO:0045444~fat cell differentiation | 4 | 0.030653 |
| GO:0050873~brown fat cell differentiation | 3 | 0.033977 |
| GO:0009409~response to cold | 3 | 0.035513 |
| GO:0071260~cellular response to mechanical stimulus | 4 | 0.040289 |
| GO:0010273~detoxification of copper ion | 2 | 0.041664 |
| GO:0043524~negative regulation of neuron apoptotic process | 5 | 0.045307 |
| GO:0071474~cellular hyperosmotic response | 2 | 0.048438 |
| GO:1903826~arginine transmembrane transport | 2 | 0.048438 |

**Table S7. KEGG analysis of of down-up mRNAs in Fed-Fasted-Refed cycle.**

| KEGG pathway | Count | *P* value |
| --- | --- | --- |
| mmu04141:Protein processing in endoplasmic reticulum | 22 | 0.000000 |
| mmu01100:Metabolic pathways | 70 | 0.000001 |
| mmu00900:Terpenoid backbone biosynthesis | 6 | 0.000192 |
| mmu00830:Retinol metabolism | 10 | 0.000570 |
| mmu01240:Biosynthesis of cofactors | 11 | 0.003952 |
| mmu05204:Chemical carcinogenesis - DNA adducts | 8 | 0.004274 |
| mmu00140:Steroid hormone biosynthesis | 8 | 0.007023 |
| mmu00520:Amino sugar and nucleotide sugar metabolism | 6 | 0.007739 |
| mmu00071:Fatty acid degradation | 6 | 0.008397 |
| mmu01250:Biosynthesis of nucleotide sugars | 5 | 0.013217 |
| mmu00590:Arachidonic acid metabolism | 7 | 0.018212 |
| mmu00982:Drug metabolism - cytochrome P450 | 6 | 0.029093 |
| mmu00980:Metabolism of xenobiotics by cytochrome P450 | 6 | 0.032294 |
| mmu03060:Protein export | 4 | 0.032689 |

**Table S8. KEGG analysis of of up-down mRNAs in Fed-Fasted-Refed cycle.**

| KEGG pathway | Count | *P* value |
| --- | --- | --- |
| mmu04068:FoxO signaling pathway | 6 | 0.005489 |
| mmu05202:Transcriptional misregulation in cancer | 7 | 0.012931 |
| mmu04380:Osteoclast differentiation | 5 | 0.024891 |
| mmu04151:PI3K-Akt signaling pathway | 8 | 0.035641 |
| mmu04150:mTOR signaling pathway | 5 | 0.047199 |

**Table S9. KEGG pathway enrichment analysis of key modules**

| Cluster | ID | Description | *P* value | Count |
| --- | --- | --- | --- | --- |
| Module1 | mmu00830 | Retinol metabolism | 0.000000 | 9 |
| Module1 | mmu00140 | Steroid hormone biosynthesis | 0.000000 | 8 |
| Module1 | mmu05204 | Chemical carcinogenesis - DNA adducts | 0.000000 | 7 |
| Module1 | mmu00591 | Linoleic acid metabolism | 0.000000 | 5 |
| Module1 | mmu00590 | Arachidonic acid metabolism | 0.000000 | 5 |
| Module1 | mmu04750 | Inflammatory mediator regulation of TRP channels | 0.000000 | 5 |
| Module1 | mmu04726 | Serotonergic synapse | 0.000008 | 4 |
| Module1 | mmu00053 | Ascorbate and aldarate metabolism | 0.000508 | 2 |
| Module1 | mmu00040 | Pentose and glucuronate interconversions | 0.000686 | 2 |
| Module1 | mmu00860 | Porphyrin metabolism | 0.000979 | 2 |
| Module1 | mmu05207 | Chemical carcinogenesis - receptor activation | 0.001625 | 3 |
| Module1 | mmu00982 | Drug metabolism - cytochrome P450 | 0.002651 | 2 |
| Module1 | mmu00980 | Metabolism of xenobiotics by cytochrome P450 | 0.002800 | 2 |
| Module1 | mmu00983 | Drug metabolism - other enzymes | 0.004410 | 2 |
| Module1 | mmu04976 | Bile secretion | 0.005190 | 2 |
| Module1 | mmu01240 | Biosynthesis of cofactors | 0.011667 | 2 |
| Module2 | mmu04622 | RIG-I-like receptor signaling pathway | 0.000000 | 4 |
| Module2 | mmu05169 | Epstein-Barr virus infection | 0.000006 | 4 |
| Module2 | mmu05162 | Measles | 0.000081 | 3 |
| Module2 | mmu05161 | Hepatitis B | 0.000112 | 3 |
| Module2 | mmu05160 | Hepatitis C | 0.000116 | 3 |
| Module2 | mmu05164 | Influenza A | 0.000134 | 3 |
| Module2 | mmu05171 | Coronavirus disease - COVID-19 | 0.000389 | 3 |
| Module2 | mmu04623 | Cytosolic DNA-sensing pathway | 0.000710 | 2 |
| Module2 | mmu05165 | Human papillomavirus infection | 0.001179 | 3 |
| Module2 | mmu04620 | Toll-like receptor signaling pathway | 0.001781 | 2 |
| Module2 | mmu05168 | Herpes simplex virus 1 infection | 0.002348 | 3 |
| Module2 | mmu04621 | NOD-like receptor signaling pathway | 0.007854 | 2 |
| Module2 | mmu05167 | Kaposi sarcoma-associated herpesvirus infection | 0.008659 | 2 |
| Module3 | mmu00900 | Terpenoid backbone biosynthesis | 0.000000 | 5 |
| Module3 | mmu00100 | Steroid biosynthesis | 0.000098 | 2 |
| Module4 | mmu04141 | Protein processing in endoplasmic reticulum | 0.000000 | 8 |
| Module4 | mmu04612 | Antigen processing and presentation | 0.000052 | 3 |
| Module4 | mmu04918 | Thyroid hormone synthesis | 0.001809 | 2 |
| Module4 | mmu05169 | Epstein-Barr virus infection | 0.016585 | 2 |
| Module4 | mmu05170 | Human immunodeficiency virus 1 infection | 0.017833 | 2 |
| Module4 | mmu05163 | Human cytomegalovirus infection | 0.020151 | 2 |
| Module5 | mmu03008 | Ribosome biogenesis in eukaryotes | 0.000533 | 2 |
| Module6 | mmu04540 | Gap junction | 0.000000 | 5 |
| Module6 | mmu04145 | Phagosome | 0.000000 | 5 |
| Module6 | mmu05132 | Salmonella infection | 0.000000 | 5 |
| Module6 | mmu05012 | Parkinson disease | 0.000000 | 5 |
| Module6 | mmu05020 | Prion disease | 0.000000 | 5 |
| Module6 | mmu05016 | Huntington disease | 0.000000 | 5 |
| Module6 | mmu05014 | Amyotrophic lateral sclerosis | 0.000000 | 5 |
| Module6 | mmu05010 | Alzheimer disease | 0.000000 | 5 |
| Module6 | mmu05022 | Pathways of neurodegeneration - multiple diseases | 0.000000 | 5 |
| Module6 | mmu04210 | Apoptosis | 0.002200 | 2 |
| Module6 | mmu04530 | Tight junction | 0.003299 | 2 |
| Module9 | mmu00561 | Glycerolipid metabolism | 0.000000 | 3 |
| Module9 | mmu00564 | Glycerophospholipid metabolism | 0.000350 | 2 |
| Module9 | mmu04936 | Alcoholic liver disease | 0.000724 | 2 |
| Module9 | mmu04150 | mTOR signaling pathway | 0.000897 | 2 |
| Module10 | mmu05322 | Systemic lupus erythematosus | 0.000000 | 4 |
| Module10 | mmu05034 | Alcoholism | 0.000000 | 4 |
| Module10 | mmu04613 | Neutrophil extracellular trap formation | 0.000000 | 4 |
| Module10 | mmu05203 | Viral carcinogenesis | 0.000064 | 3 |
| Module12 | mmu00982 | Drug metabolism - cytochrome P450 | 0.000000 | 4 |
| Module12 | mmu00980 | Metabolism of xenobiotics by cytochrome P450 | 0.000000 | 4 |
| Module12 | mmu00350 | Tyrosine metabolism | 0.000115 | 2 |
| Module12 | mmu00620 | Pyruvate metabolism | 0.000139 | 2 |
| Module12 | mmu00071 | Fatty acid degradation | 0.000195 | 2 |
| Module12 | mmu00010 | Glycolysis / Gluconeogenesis | 0.000324 | 2 |
| Module12 | mmu00480 | Glutathione metabolism | 0.000375 | 2 |
| Module12 | mmu01524 | Platinum drug resistance | 0.000463 | 2 |
| Module12 | mmu05204 | Chemical carcinogenesis - DNA adducts | 0.000510 | 2 |
| Module12 | mmu00983 | Drug metabolism - other enzymes | 0.000612 | 2 |
| Module12 | mmu00830 | Retinol metabolism | 0.000680 | 2 |
| Module12 | mmu04936 | Alcoholic liver disease | 0.001432 | 2 |
| Module12 | mmu05418 | Fluid shear stress and atherosclerosis | 0.001556 | 2 |
| Module12 | mmu05225 | Hepatocellular carcinoma | 0.002174 | 2 |
| Module12 | mmu05208 | Chemical carcinogenesis - reactive oxygen species | 0.003517 | 2 |
| Module12 | mmu05207 | Chemical carcinogenesis - receptor activation | 0.003612 | 2 |
| Module13 | mmu00360 | Phenylalanine metabolism | 0.000000 | 3 |
| Module13 | mmu00350 | Tyrosine metabolism | 0.000000 | 3 |
| Module13 | mmu00380 | Tryptophan metabolism | 0.000195 | 2 |
| Module13 | mmu00270 | Cysteine and methionine metabolism | 0.000210 | 2 |
